# Large-Scale Evolutionary Analysis of Genes and Supergene Clusters from Terpenoid Modular Pathways Provides Insights into Metabolic Diversification in Flowering Plants

**DOI:** 10.1371/journal.pone.0128808

**Published:** 2015-06-05

**Authors:** Johannes A. Hofberger, Aldana M. Ramirez, Erik van den Bergh, Xinguang Zhu, Harro J. Bouwmeester, Robert C. Schuurink, M. Eric Schranz

**Affiliations:** 1 Wageningen University and Research Center, Droevendaalsesteeg 1, 6708 PB, Wageningen, The Netherlands; 2 Chinese Academy of Sciences/Max Planck Partner Institute for Computational Biology, 320 Yueyang Road, Shanghai, 200031, PR China; 3 University of Amsterdam, Swammerdam Institute for Life Sciences, Science Park 904, 1098 XH, Amsterdam, The Netherlands; University of Copenhagen, DENMARK

## Abstract

An important component of plant evolution is the plethora of pathways producing more than 200,000 biochemically diverse specialized metabolites with pharmacological, nutritional and ecological significance. To unravel dynamics underlying metabolic diversification, it is critical to determine lineage-specific gene family expansion in a phylogenomics framework. However, robust functional annotation is often only available for core enzymes catalyzing committed reaction steps within few model systems. In a genome informatics approach, we extracted information from early-draft gene-space assemblies and non-redundant transcriptomes to identify protein families involved in isoprenoid biosynthesis. Isoprenoids comprise terpenoids with various roles in plant-environment interaction, such as pollinator attraction or pathogen defense. Combining lines of evidence provided by synteny, sequence homology and Hidden-Markov-Modelling, we screened 17 genomes including 12 major crops and found evidence for 1,904 proteins associated with terpenoid biosynthesis. Our terpenoid genes set contains evidence for 840 core terpene-synthases and 338 triterpene-specific synthases. We further identified 190 prenyltransferases, 39 isopentenyl-diphosphate isomerases as well as 278 and 219 proteins involved in mevalonate and methylerithrol pathways, respectively. Assessing the impact of gene and genome duplication to lineage-specific terpenoid pathway expansion, we illustrated key events underlying terpenoid metabolic diversification within 250 million years of flowering plant radiation. By quantifying Angiosperm-wide versatility and phylogenetic relationships of pleiotropic gene families in terpenoid modular pathways, our analysis offers significant insight into evolutionary dynamics underlying diversification of plant secondary metabolism. Furthermore, our data provide a blueprint for future efforts to identify and more rapidly clone terpenoid biosynthetic genes from any plant species.

## Introduction

To elucidate the dynamics underlying metabolic diversification across multiple lineages, it is of paramount importance to identify and distinguish the complete set of orthologous and paralogous loci present within multiple genome annotations in a phylogenetic framework [1]. Two homologous genes are referred to as orthologs if they descend from one locus present in the common ancestor lineage and split due to speciation [2,3]. By definition, orthologous genes are embedded in chromosomal segments derived from the same ancestral genomic locus, thus sharing high inter-species synteny between closely related lineages [4]. In contrast, paralogous loci refer to homologs within one lineage and are due to, for example, tandem-, transposition- or whole genome duplications (WGDs) [5,6]. Large-scale synteny is not observed for paralogs derived from small-scale events like tandem- and transposition duplication. In contrast, paralogs derived from WGDs are located within intra-species syntenic genomic blocks, and can be referred to as ohnologs or syntelogs [7,8]. Supergene loci refer to clusters of genes in close genomic proximity, often causing linkage disequilibrium [9,10]. Tandem duplicates comprise arrays of paralog supergenes that are due to, for example, errors in meiosis like unequal crossing over and have been connected to metabolic diversification in plants [11–13].

Together with the continuous progress and use of next generation sequencing techniques, genome-wide analysis of syntelog distribution provided evidence for a history of ancient shared and/or lineage-specific polyploidy events for all flowering plant lineages [4]. For example, the lineage of the model plant *Arabidopsis* underwent at least five polyploidy events during evolution, two preceding and three following angiosperm evolution [14]. Among those, the most recent WGD event is commonly referred to as “At-α” and is shared by all other mustard family members, including the extant sister clade of the Aethionemeae [15,16]. The more ancient At-β WGD event is in turn shared by core species in the order Brassicales, and excepting early-branching lineages such as papaya [17,18] and therefore occurred after split of the *Carica* lineage. At-y refers to an older whole genome triplication (WGT) event with evidence in all Asterids (including tomato) and Rosids, grape (Vitales) and basal clades such as *Pachysandra terminalis* (Buxales) and *Gunnera manicata* (Gunnerales) [19,20]. Crops like *Brassica rapa* (Br-α WGT), *Solanum lycopersicum* and *Solanum tuberosum* (Sol-α WGD/WGT) also show evidence of ancient genome multiplications [21,22]. As a consequence, the level of “genome multiplicity” expected from the successive WGDs/WGTs in *B*. *rapa* (defined as “syntenic depth”) is 36x when compared to the 1x eudicot ancestor (3x due to At- y, 2x due to At-β, another 2x due to At-α as well as 3x due to Br- α, see above).

Evidence is now accumulating for significant impact of ancient and recent gene and genome duplication events to birth and diversification of key biological traits. Duplication was proposed to be a key factor in expansion of regulatory and enzymatic pathways involved in generation of >200,000 diverse biochemical secondary metabolites in the flowering plant lineage [23–25]. For example, a differential impact of various duplication modes has been revealed for plant resistance proteins [26]. Likewise, the last three polyploidy events of the *Arabidopsis*-lineage (see above) likely contributed to shaping the genetic versatility of the glucosinolate pathway, a class of plant secondary metabolites with beneficial effects to human health and nutrition [25]. Similarly, polyploidy has been brought in connection to the origin of C4-photosynthesis in Cleomaceae [27].

Little is known about the impact of genome duplication to diversification of isoprenoid pathways. Isoprenoids form a highly diverse class of metabolites commonly found in all kingdoms of life [28]. In angiosperms, for example, phytol side-chain substitutes of chlorophyll and carotenoid pigments as well as phytohormones like gibberellin or brassinosteroids are well-characterized isoprenoids involved in basic metabolic processes that are essential for plant growth and development [29–31]. To the knowledge of the authors, evidence for the connection of polyploidy to secondary metabolite pathway evolution is to date only available for glucosinolates biosynthesis in the mustard family [25]. Note that both glucosinolates and terpenoids are defined as specialized or secondary metabolites that play major roles in plant-insect interactions like, for example, attraction of beneficial organisms or defense against herbivores [32]. Boutanaev et al. investigated core terpene synthase (*TPS*) genes (which generate terpene scaffold diversity) and identified micro-syntenic clusters that have arisen within recent evolutionary history by gene duplication, acquisition of new function and genome reorganization [33]. Note that in concert with *TPS* genes, terpenoid biosynthesis depends on various independent pathways (referred to as modules hereafter). Here, we performed further extended comparative analysis of various independent terpenoid biosynthetic modules in context of gene- and genome duplication.

**Fig 1:**
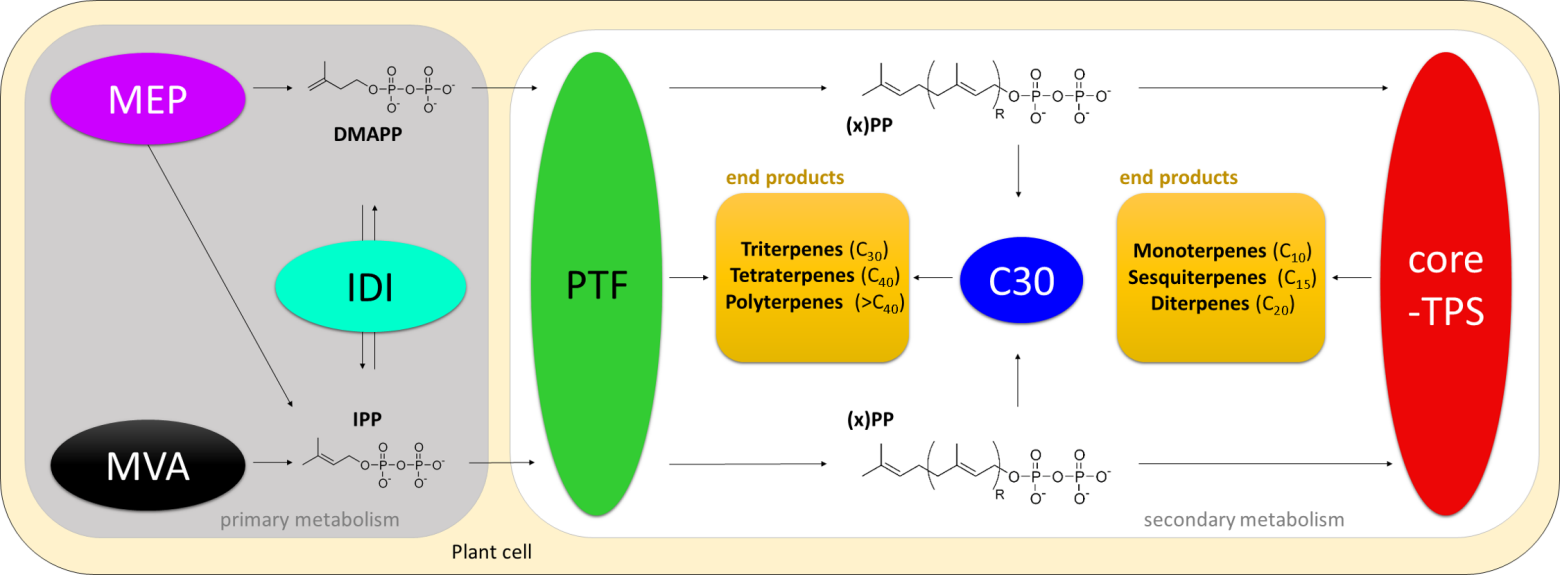
Overview of all plant specialized terpenoid biosynthetic modules. Proteins involved in the mevalonic (MVA, shown in black) and methylerythritol phosphate (MEP, shown in purple) pathways synthesize the universal C_5_-isoprenoid building blocks isopentenyl diphosphate (IPP) and its isomer dimethylallyl diphosphate (DMAPP). Those compounds can be isomerized using enzymes of the IPP isomerase class (IDI, shown in turquois). Subsequently, the C_5_- blocks are transferred by enzymes of the prenyltransferase (PTF, shown in green) group to the isoprenoid intermediates with variable carbon backbone chain lengths (i.e. C_10_ for geranyl pyrophosphate; C_15_ for farnesyl pyrophosphate; C_20_ for geranylgeranyl phosphate and nerolidol diphosphate). Terpene synthase (core-*TPS*) gene products (shown in red) further catalyze biosynthesis of C_10_ (mono-), C_15_ (sesqui-) or C_20_ (di-) terpenes (end products, shown in yellow). C30 (shown in blue) refers to enzymes catalyzing biosynthesis of specific triterpenes (end products, shown in yellow). Likewise, prenyltransferases are involved in biosynthesis of longer-branched tetra- and polyterpenes (shown in green).

Briefly, a sequential combination of six distinct reaction modules acts in concert to convert primary metabolites to longer-chain compounds mediating designated biological function. Therefore, plant terpenoid biosynthesis displays “modular” organization, including (1) *TPS* genes, (2) IPP isomerases (IDI), (3) prenyltransferases (PTF), (4) genes from MVA and (5) MEP pathways as well as (6) triterpene-specific synthases (see **Fig 1**for a comprehensive overview). Notably, genes involved in the latter three modules share a common evolutionary origin (i.e. genes are homologous) as previously described based on analyses of Solanaceae species [34]. All terpenoids are synthesized from two universal C_5_-isoprenoid building blocks (a) isopentenyl diphosphate (IPP) and (b) its isomer dimethylallyl diphosphate (DMAPP). In plants, IPP is synthesized independently by the mevalonate (MVA, shown in black in **Fig 1**) and methylerythritol phosphate (MEP, Shown in purple in **Fig 1**) pathways. In contrast, DMAPP is synthesized by enzymes of the MEP pathway only [35]. Both DMAPP and IPP compounds can be isomerized by enzymes of the IPP isomerase type (IDI, shown in turquois in **Fig 1**) [36]. Due to the economic relevance of enzymes involved in MEP and MVA pathways as well as IPP isomerases, the underlying biochemistry has been thoroughly investigated. Note that both MVA and MEP pathways comprise sequential arrangements of consecutive reaction steps leading to formation of intermediate products [37]. Analysis of stoichiometry indicated dosage-dependent effects regarding both pathways in yeast [38]. Going beyond yeast, comparative network analysis of MVA and MEP pathways in prokaryotes and the model plant *A*. *thaliana* characterized dosage-dependent effects of enzymes in both pathways and elevations of corresponding metabolite concentrations in plants and humans. This indicates that enzymes involved in MVA and MEP pathways operate concentration-dependent across all kingdoms of life [37,39]. Similarly, genetic engineering of *Escherichia coli* in context of industrial terpenoid production revealed that enzymes of the IDI group function in a dosage-dependent manner [40]. This was confirmed by mechanistic investigations of IDI enzymes in *Thermus thermophiles* due to their relevance for a wide range of biotechnological applications [41]. Likewise, dosage-dependent effects have been revealed for plant-derived IDI enzymes. For example, the economic potential of *in vitro* production of caoutchouc led to cloning, heterologous expression and functional characterization (i.e. determination of biochemical function) of *IDI* loci from the rubber tree *Hevea brasiliensis* [42].

Enzymes of the prenyltransferase class (PTF, shown in green in **Fig 1**) subsequently catalyze formation of C_10_-prenyl diphosphate molecules. Moreover, they can mediate the (optional) elongation of the C_10_-backbone by the addition of further C_5_-isopentenyl diphosphate units necessary for formation of di- and sesquiterpenes including longer-chain (C_25_-C_55_) tetra- and polyterpenes [43–45]. Terpene synthases (encoded by *TPS* genes, shown in red in **Fig 1**) catalyze conversion of specific C_10_-, C_15_ or C_20_ isoprenoid precursors to specialized monoterpenes (C_10_), sesquiterpenes (C_15_) and diterpenes (C_20_), building a module further downstream within terpenoid biosynthesis, respectively [29,46]. Specialized triterpene synthases catalyze formation of pentacyclic triterpenes (such as lupane and squalene) (C_30,_ shown in blue in **Fig 1**) [47–49]. Note that those compounds can be further modified in distant branches of plant secondary metabolism, for example to triterpene alcohols (such as lanosterol and cycloarthenol) with various bioactivities [50,51]. Entry to the aforementioned MEP pathway was previously proposed to be catalyzed by two divergent 1-deoxy-D-xylulose 5-phosphate synthase isoforms in *S*. *lycopersicum* (Sl*DXS1* / Sl*DXS2*) and *A*. *thaliana* (At*DXS1* / At*DXS2*) [28,52–54]. In tomato, *DXS1* is ubiquitously expressed whereas *DXS2* transcripts are abundant in a few tissue types including glandular trichomes. Trichomes are hair-like structures present in the aerial parts of many plant species. They exhibit tremendous diversity but are of general interest to plant breeders since they are often responsible for the production of plant secondary metabolites with various bioactivities, including terpenoids [55–57]. Interestingly, knock-down of *DXS2* led to a differential distribution of mono- and sesquiterpenes within tomato glandular trichomes as well as to a significant increase of trichome density, giving rise to economic and ecological potential to this small gene family [52].

The core-*TPS* gene family has been most intensively studied in the model plant *Arabidopsis thaliana*. The ecotype Col-0, that contains 32 full-length functional and 8 pseudogenes of the terpene synthase type, of which about a third have been annotated to a designated biochemical function by functional characterization [29,58–64]. Most of the Col-0 core-*TPS* genes are constitutively expressed in roots, flowers or leaves for production of mono-, di- or sesquiterpenes whereas some are up-regulated under presence of specific stress-related stimuli [58,64,65]. Notably, 27 of the 32 Col-0 core-*TPS* genes comprise supergene clusters organized in 16 tandem arrays [11], whereas two of them constitute an ohnolog duplicate gene pair due to the most recent At-α ancient whole genome duplication event (see above) [8]. Beyond *A*. *thaliana*, efforts to identify core-*TPS* genes have been published for tomato (*S*. *lycopersicum*) [66], orange (*C*. *sinensis*) [67], eucalyptus (*E*. *grandis*) [68], grape (*V*. *vinifera*) [69], millet (*S*. *bicolor*), apple (*M*. *domesticus*) [70] and the basal Angiosperm *Amborella* (*A*. *trichopoda*) [71]. However, functional characterization (i.e. distinct biochemical function) of all *TPS* genes present within a species are currently available for tomato and the model plant *Arabidopsis* only. The complete set of biosynthetic elements involved in both MEP and MVA pathways as well as other terpenoid-associated prenyltransferases including triterpene-specific synthases has to-date only been described in *Arabidopsis* with a total of 34 genes [35]. Among those, 15 possess prenyltransferase activity, whereas nine and eight belong to the MVA and MEP pathway, respectively. Furthermore, the *A*. *thaliana* genome contains two genes encoding proteins of the IPP isomerase (IDI) type with similar bioactivities [35,36]. In total, the gene count of all modules within the complete terpenoid biosynthetic pathway therefore rises to 66 including 32 functional core-*TPS* genes in *Arabidopsis*, with a 64% (42 / 66) fraction of tandem duplicate supergenes and 21% (14 / 66) comprising ohnolog duplicate gene pairs dating back to the At-α, At-γ or At-β ancient whole genome multiplication events (see above) [8,11,72].

In this study, we employed a meta-method by combining evidence provided by sequence homology (BLAST), HMM Modelling (interpro scan) and genomic context (SynMap) for robust annotation of genes involved in all modules of terpenoid biosynthesis on a uniquely broad phylogenomics framework. First, we infer novel annotation for loci previously not brought in connection to terpenoid biosynthesis within 17 genome assembly including twelve major crops, thereby providing insights to diversification of plant secondary metabolism during 250 MA of flowering plant evolution. Second, we assessed and compared key factors contributing to copy number variation across all terpenoid biosynthetic modules, thereby providing evidence for the impact of gene- and genome duplication to metabolic diversification in plants. Third, we established a novel clade of duplicate genes with pleiotropic effects in control of trichome density and terpenoid biosynthesis, thereby providing data that support the concept of functional divergence following gene and genome duplication. In summary, our data offer significant insight into evolutionary dynamics underlying diversification of plant secondary metabolism. Furthermore, we provide a blueprint for future efforts to identify and more rapidly modify terpenoid biosynthetic genes across all modules in any flowering plant species.

## Materials and Methods

### Software prerequisites

All employed Perl and Python scripts required perl (strawberry v5.18) and Python (v2.7) libraries including Bioperl (v1.6.910) and Biopython (v1.63) modules, respectively. The iprscan_urllib.py-script for HMM-based domain annotation (see below) required SOAPy, NumPy and urllib Python modules. For BLAST screens, we employed the stand-alone command line version of NCBI BLAST 2.2.27+ (ftp://ftp.ncbi.nlm.nih.gov/blast/executables/blast+/LATEST/, last accessed on December 13th, 2014) [73]. Fisher’s exact test for count data was performed using the R package for statistical computing (www.r-project.org, last accessed on December 13th, 2014).

### Genome annotations

In total, we analyzed 15 draft genomes as well as two gene-space assemblies represented by non-redundant transcriptomes. The complete sets of representative genes and proteins for 12 of these 17 datasets were downloaded using www.phytozome.net, last accessed on December 13th, 2014) [74] and the CoGe package for comparative genomics [4]. We included *Amborella trichopoda* EVM27 [71], *Arabidopsis thaliana* TAIR10 [75], *Brassica rapa* v1.1 [76], *Carica papaya* v0.5 [18], *Citrus sinensis* v1 [77], *Eucalyptus grandis* v1.1 [78], *Glycine max* Wm82.a2.v1 [79], *Sorghum bicolor* v1.4 [80], *Solanum tuberosum* v3.2.10 [81], *Solanum lycopersicum* v2.40 (Potato Genome Consortium 2012), *Vitis vinifera* Genoscope.12X [20] and *Zea mays* 5a.59 [82]. *Tarenaya hasslerania* v5 [83] (Weber, Schranz et al, unpublished data), *Cleome gynandra* v2 (Weber, Schranz et al. 2014, unpublished data) and *Nicotiana benthamiana* v0.42 [84] genome annotations were made available by the authors. Non-redundant transcriptomes of *Cannabis sativa* ChemDawg (marijuana) [85] and *Lactuca sativa* (Mitchelmoore et al, unpublished data) early-draft gene-space assembly of were extracted from Genbank [86].

### *De novo* protein annotation of early-draft gene-space assemblies

Non-redundant transcriptome data of *Cannabis sativa* ChemDawg (marijuana) and *Lactuca sativa* derive from unpublished early-draft gene-space assembly (see above) and therefore contain significant parts of non-coding sequences as well as putative sequencing errors. We therefore subjected both datasets to the translatedna.py script v1.75 (https://github.com/jenhantao/HiSeq/blob/master/translatedna.py, last accessed on December 13th, 2014). First, single mRNA sequences were translated in all six frames. Second, the peptide fragment encoded by the largest open reading frame was printed. All other parts were discarded. The output comprises an approximation on the non-redundant set of proteins for both species.

### Confirmation and expansion of multi-gene families associated with the terpenoid biosynthetic module in *Arabidopsis thaliana* (“run 1”)

Functional annotation of target genes across all organisms was an interlaced approach consisting of 3 independent BLAST screens (run 1–3). For run 1 in *A*. *thaliana*, we obtained 32 core-*TPS* genes from [29] as well as 34 genes acting in modules further up- and downstream in terpenoid biosynthesis [35]. We queried all 66 sequences against the TAIR10 *A*. *thaliana* genome annotation in a BLAST screen without e-value threshold (forward run). We extracted all target sequences and queried them back against the *A*. *thaliana* TAIR10 genome annotation with an applied target sequence maximum threshold of 2 (under consideration of self-hits produced by Col-0 genes within this pool) (reverse run). After removal of self-hits, we scored loci as associated with the *A*. *thalian*a terpenoid specialized metabolism if they were part of the target sequence pool in the forward run, and aligned to a terpenoid biosynthetic gene as defined by [29,35] in the reverse run. We thereby created an extended set of *A*. *thaliana* TP-associated loci (85 genes).

### Species-wise determination of putative homologous gene anchors (“run 2”)

In the next step for large-scale specialized terpenoid biosynthetic gene identification, run 2 determined unidirectional best BLAST hits for both (a) protein and (b) coding DNA sequences between *A*. *thaliana* Col-0 and all other 14 genome annotations in a screen without e-value thresholds (for early-draft gene-space assemblies, only protein data were used). Since terpenoid biosynthetic loci can comprise multiple domain types connected by partially conserved linkers, the BLAST approach can result in false positives due to short but highly conserved highest-scoring sequence pairs (HSPs) in functionally non-relevant (i.e. structural) parts of the protein. Therefore, we developed a python script to discard target sequences with a query/target sequence length ratio below 0.5 and above 2.0 as previously described to avoid false positive BLAST results due to short but highly conserved highest-scoring sequence pairs (HSPs) in functionally non-relevant (i.e. structural) parts of the protein [26]. We determined (c) additional, length-filtered HSP pairs (based on both CDS and proteins) for these loci within the aforementioned length ratio scope to form a 2nd line of evidence for homolog gene detection as previously described [26].

### Syntelog / ohnolog determination

Calculation of pairwise syntenic blocks within and between genomes is based on integer programming [87] but implemented to an easy-to-use web interface termed CoGe package for comparative genomics (www.genomevolution.org, last accessed on December 13th, 2014) [4,88]. Within all genome assemblies, we determined genes sharing the same genomic context to counterparts in the *A*. *thaliana* Col-0 genome annotation (defined as syntelogs) using the DAGchainer [89] and Quota-Align [87] algorithms implemented to the “SynMap” function within CoGe. To mask noise generated by successive duplication(s) of ohnolog blocks including segmental duplications, we applied Quota-Align ratios for the “coverage depth”-parameter that are consistent with the syntenic depth (defined as the level of “genome multiplicity” expected from the multiplication of successive WGDs/WGTs) calculated for each genome annotation. For merging of adjacent syntenic blocks, we applied a threshold of n = 350 gene spacers. For within-species ohnolog counterparts of target genes, we applied the “Synfind” function within the CoGe package (https://genomeevolution.org/CoGe/SynFind.pl, last accessed on December 13th, 2014). To decrease false-positive scoring of recent segmental duplications, we set maximum threshold values of 1.5 for the Ks-value averages between duplicate gene copies. This facilitates selective scoring of ohnolog duplicate pairs within genomic blocks that are due to polyploidy as previously described [4]. Please note that we appended URLs to regenerate genome-wide ohnolog identification for 13 out of 17 genomes subjected to this analysis (see Results section).

### Determination of tandem duplicate gene copies

Following a widely-used method for tandem duplicate identification, we queried the complete set of proteins encoded in the whole genome assembly against itself in a BLAST screen without any e-value threshold (this ensures the identification of most homologs including highly diverged ones) and filtered our final set of target sequences from above outside a window of n = 10 allowed gene spacers in both directions from the query sequences (this ensures the identification of adjacent duplicates organized in arrays among all homologs scored above) as previously described for the identification of tandem duplicates [11]. We acknowledged that determination of genome-wide tandem duplicate frequencies following this approach decreases in accuracy with increased degrees of assembly fragmentation (i.e. total number of scaffolds/contigs). This means that false-negatives singletons are more likely scored in genomes with many short scaffolds (“gene-space assemblies”) compared to annotations with few scaffolds in the size-range of chromosome pseudo-molecules which is due to the lack of information on the relative order of scaffolds. Similarly, it is not possible to score tandem duplicates based on non-redundant transcriptomes because those represent collections of single transcripts without information of the genomic context. As a result, our analysis of tandem duplicate fractions was restricted to 13 genome assemblies.

### Scoring of putative gene transposition duplicate pairs among *Arabidopsis DXS*-like genes

Scoring of gene transposition duplicate pairs among *DXS* genes involved three steps. First, we obtained all tandem- and ohnolog duplicates present within the gene family as described above. Second, we queried CDS sequences of non-tandem/non-ohnolog duplicate target genes against the *Arabidopsis* genome in a BLAST screen without e-value threshold. Third, we generated (B)LastZ two-way alignments of the genomic regions that harbor (a) query as well as (b) highest-scoring non-self target sequence within a 40 kb window (20 kb on each side). This was accomplished using the GEvo function from the CoGe comparative genomics package (http://genomevolution.org/CoGe/GEvo.pl, last accessed on December 13^**th**^, 2014) [4]. Graphical highlights of transposon-like sequences have been customized by choosing “show other features” in the “results visualization” tab. We scored *DXS*-like gene pairs as gene transposition duplicates if they comprise highest-scoring sequence pairs embedded in otherwise non-syntenic regions, while both loci showing evidence for adjacent fragments of transposable elements as previously described [25].

### Determination of anchor paralogs and generation of extended multi-gene family pools across all analyzed species (“run 3”)

Since ortholog detection based on unidirectional or reciprocal best BLAST hits can miss many “real” orthologs in duplicate-rich species like animals or plants [90], a separate run was necessary to increase accuracy. For run 3, we defined the initial homologous genes set as the merged set consisting of five HSP partner groups (first group: based on length-filtered protein pairs; second group: based on non-length-filtered protein pairs; third group: based on non-length-filtered CDS pairs; fourth group: based on length-filtered CDS pairs; fifth group: based on syntelogs, see above for length filter criteria). We thereby created a set of putative homologous loci anchoring all *A*. *thaliana* gene families in all other analyzed genome annotations (“anchor pool”). In a next step, we performed a BLAST search without e-value thresholds to query all homologous anchor genes against all 17 genomes in a species-wise manner to determine putative paralogs of the anchor gene set (“run 3 forward”). We extracted all target sequences and queried them against the *A*. *thaliana* Col-0 TAIR10 genome annotation with a target sequence maximum threshold of 2 (“run 3 reverse”). After removal of self-hits, we scored loci as associated with terpenoid biosynthesis within their species if they align to any member of the extended terpenoid biosynthetic loci in *A*. *thaliana* (see above). We defined all members of this pool as homologous to the anchor pool if they were not present within the set of homologous anchor genes (see above).

### Hidden Markov Modeling and prediction of protein domains

Since we included highest-scoring sequence partners based on BLAST as well as syntelogs, the above-mentioned extended multi-gene family pool of terpenoid biosynthetic genes is based on both sequence homology and genomic location of its members. However, we observed an erosion of synteny across lineages relative to their phylogenetic distance. Furthermore, DNA sequence homology decreases with phylogenetic distance due to wobble rules for the 3^rd^ codon position. Likewise, the protein sequence homology between distant multi-gene family members can decrease due to synonymous substitutions of amino acids belonging to the same chemical class (i.e. aliphatic, aromatic, basic, cyclic). Therefore, we applied a final filtering step to remove false-positive loci from the extended terpenoid biosynthetic genes pool across all genomes (including the extended terpenoid biosynthetic genes pool in *Arabidopsis*, see above). Using the iprscan_urllib.py script provided by the European Molecular Biology Laboratory (EMBL, Heidelberg, Germany) (https://www.ebi.ac.uk/Tools/webservices/download_clients/python/urllib/iprscan_urllib2.py, last accessed on December 13th, 2014), we queried every member of the terpenoid biosynthetic genes pool (including the extended set determined for *A*. *thaliana*, see above) to 14 algorithms that apply Hidden Markov Models for (protein domain) signature recognition (BlastProDom, FPrintScan, HMMPIR, HMMPfam, HMMSmart, HMMTigr, ProfileScan, HAMAP, PatternScan, SuperFamily, SignalPHMM, TMHMM, HMMPanther and Gene3D) [91]. We overcame the one-sequence-at-a-time limitation of the EMBL server by writing batch wrappers for 25x-fold parallelization. As a result, we mapped all protein domains present in the putative multi-gene family pool onto their genes in less than a day, and discarded all false positive genes from the whole set (i.e. genes not encoding at least one domain common to at least one reaction module). Referencing of all identified genes to distinct terpenoid biosynthetic modules was based on presence of module-specific protein domains.

### Multiple protein alignments

To generate multiple alignments of protein sequences, the stand-alone 64-bit version of MAFFT v7 was employed (http://mafft.cbrc.jp/alignment/software/, last accessed on December 13th, 2014) [92]. First, all terpenoid biosynthetic proteins were aligned species-wise using the command line {mafft.bat—anysymbol—thread 4—threadit 0—reorder—auto input > output} Mesquite v2.75 (http://mesquiteproject.org, last accessed on December 13th, 2014) was used with multi-core preferences to trim MAFFT multiple alignments down to gap-free sites. Trimmed blocks were re-aligned using MAFFT with the command line {mafft.bat—anysymbol—thread 4—threadit 0—reorder—maxiterate 1000—retree 1 –localpair input > output}.

### Microarray-based gene expression analysis in *Arabidopsis*

To test differential and trichome-specific expression of *DXS*-like genes in *Arabidopsis*, we have used a Col-0 wild type trichome-specific transcriptome dataset (available at the TrichOME database, http://www.planttrichome.org/, last accessed on December 13th, 2014) [93]. Normalized values of three independent experiments performed with the ATH1 microarray were generated and averaged as described [94]. For calculation of relative gene expression, we referenced the bHLH-motif containing house-keeping gene AT4G34720 [95].

### Quantitative PCR-based gene expression analysis in tomato (*S*. *lycopersicum*)

Leaves, stems and roots were collected in triplicate from 4-week-old *Solanum lycopersicum* cultivar Moneymaker plants. Part of the stems were left intact and part were used for trichome isolation by shaking the stems in liquid nitrogen. Frozen isolated trichomes, stems that remained after trichome removal, intact stems, leaves and roots were ground to a fine powder and subjected to RNA isolation with Tri Reagent (Sigma) and DNase treatment (TURBO DNase, Ambion) according to the manufacturer’s instructions. cDNA was synthesized from 1 μg of total RNA using the RevertAid kit (Fermentas). RT-qPCR was used to study the expression of 1-deoxy-d-xylulose 5-phosphate synthase isoforms 1, 2 and 3 (*DXS1*, *DXS2*, and *DXS3*) in cDNA derived from different tissues. Gene specific primers were designed using Primer3Plus (*DXS1*-F: 5’-ATTGGGATATGGCTCAGCAG-3’; *DXS1*-R: 5’-CAGTGGTTTGCAGAAACGTG-3’; *DXS2*-F: 5’-TTTACCGACCGCAACCTTAG-3’; *DXS2*-R: 5’-GTGCTTGAGGTCCAATTTGC-3’; *DXS3*-F: 5’- AATGGAGCCTTCACTTCACC-3’; *DXS3*-R: 5’-ACCCAGCTGCAAATGTTACC-3’). Tomato RUB1 conjugating enzyme-encoding (*RCE1*) gene (Gen-Bank accession no. AY004247) (*RCE*-F: 5’- GATTCTCTCTCATCAATCAATTCG-3’ and *RCE*-R ‘5-GAACGTAAATGTGCCACCCATA-3’) was used for normalization. PCR reactions were prepared in duplicate by mixing cDNA equivalents of 10 ng RNA with the SYBR Green Real-Time PCR master mix (Invitrogen) and 300 nM of each primer. Quantification of the transcript level was performed in an ABI 7500 Real-Time PCR System (Applied Biosystems) with the following cycling program: 2 min, 50°C, 15 min 95°C, 45 cycles of 15 sec at 95°C and 1 min at 60°C followed by a melting curve analysis. At the end of each run, amplified products were sequenced to verify their identity. Relative expression values were calculated using the efficiency δCt method as previously described [96]. All wet-lab expression analysis were performed in four independent biological and three technical replicates.

### Phylogenetic and similarity/identity analysis

We performed Bayesian Markov chain Monte Carlo (MCMC) analysis using MrBayes version 3.2.2 (http://mrbayes.sourceforge.net/, last accessed on December 13th, 2014) [97] with the following parameters: Dirichlet model; uniform gamma shape parameter variation 0.00–200.00; 50 million generations; 2 independent runs, 4 chains each; temperature heating 0.2; sample taking every 5000 generations; burn-in time at 12500000 samples. Bayesian inference trees were constructed using the CIPRES package (http://www.phylo.org/sub_sections/portal/, last accessed on December 13th, 2014) [98]. Model convergence was checked in Tracer version 1.5 (http://tree.bio.ed.ac.uk/software/tracer/, last accessed on December 13th, 2014) [99]. FigTree v1.3.1 was used to generate and edit phylogenetic trees (http://tree.bio.ed.ac.uk/software/figtree/, last accessed on December 13th, 2014) [100]. Results were scored reliable once the effective sampling size of all parameters was above 100. Tree branches supported with posterior probabilities (PP) below 0.7 were considered weak and above 0.9 as strong. Protein sequence similarity analysis were performed using the Needle program from the EMBOSS software package (http://emboss.sourceforge.net/, last accessed on December 13th, 2014) [101].

### Ethics Statement

The authors hereby state that no specific permissions were required for any activities and/or locations that are connected to this research. Likewise, the authors hereby confirm that the research summarized in this article did not involve endangered or protected species. In addition, the authors hereby state clearly that all sampling procedures and/or experimental manipulations were reviewed/specifically approved and no field permit was required. Wageningen University & Research Center and all other institutes affiliated with this work comprise legal entities that do not act on any basis that is prohibited by local, state or federal law.

## Results

### (Re)annotation of the *Arabidopsis* terpenoid biosynthetic inventory and expansion of genes associated with all reaction modules

As an initial step for identification of genes involved in all terpenoid biosynthetic modules, we reviewed current literature to pool all published *Arabidopsis* core-TPS genes with biosynthetic elements acting further up- and downstream in the pathway (**Fig 1**) [29,35,61]. As a result, we generated a list of 66 biosynthetic elements previously identified in the model plant (**Table 1**). This compilation represents a patchwork of information containing both genes found by functional studies as well as genes with computationally inferred association to terpenoid metabolism. Hence, uniform standards of gene identification have not been applied for curation of this initial list.

**Table 1:** The published terpenoid biosynthetic module in *Arabidopsis*.

Gene ID	Annotation	Tandem duplicate	Bowers pair^B^	Reference^C^
*Isopentenyl diphosphate (IPP) isomerases*				
AT3G02780	*IDI1*	-	A12N076	Campbell et al., 1998
AT5G16440	*IDI2*	-	A12N076	Campbell et al., 1998
*Mevalonic acid (MVA) pathway*				
AT1G31910	*HMG1*	Yes	-	Benveniste et al., 2002
AT1G76490	*PMK*	Yes	C2N120	Caelles et al., 1989
AT2G17370	*HMG2*	-	C2N120	Caelles et al., 1989
AT2G38700	*MVD1*	-	A11N067	Cordier et al., 1999
AT3G54250	*MVD2*	-	A11N067	Benveniste et al., 2002
AT4G11820	*HMGS*	-	-	Montamat et al., 1995
AT5G27450	*MK*	-	-	Riou et al., 1994
AT5G47720	*ACT1*	Yes	-	Ahumada et al., 2008
AT5G48230	*ACT2*	Yes	-	Ahumada et al., 2008
*Methylerythritol phosphate (MEP) pathway*				
AT1G63970	*MDS*	-	-	Hsieh and Goodman, 2006
AT2G02500	*MCT*	-	-	Rohdich et al., 2000
AT2G26930	*CMK*	-	-	Hsieh et al., 2008
AT4G15560	*DXS1*	-	A15N013	Lange et al., 2003
AT4G34350	*HDR*	-	-	Hsieh and Goodman, 2005
AT5G11380^D^	*DXS3*	-	-	Lange et al., 2003
AT5G60600	*HDS*	-	-	Rodríguez-Concepción et al., 2002
AT5G62790	*DXR*	-	-	Schwender et al., 1999
*Prenyltransferases (PTF)*				
AT1G49530	*GGPS1*	Yes	-	Zhu et al., 1997a
AT2G18620	*GGPS2*	Yes	A10N118	Wang and Dixon, 2009
AT2G18640	*GGPS3*	Yes	-	Okada et al., 2000
AT2G23800	*GGPS4*	Yes	A10N309	Zhu et al., 1997b
AT2G34630	*GPS1*	-	-	Bouvier et al., 2000
AT3G14510	*GGPS5*	Yes	-	Finkelstein et al., 2002
AT3G14530	*GGPS6*	Yes	-	Wang and Dixon, 2009
AT3G14550	*GGPS7*	Yes	-	Okada et al., 2000
AT3G20160	*GGPS8*	-	-	Zhu et al., 1997a
AT3G29430	*GGPS9*	Yes	-	Finkelstein et al., 2002
AT3G32040	*GGPS10*	Yes	-	Finkelstein et al., 2002
AT4G17190	*FPS1*	Yes	A21N001	Cunillera et al., 2000
AT4G36810	*GGPS11*	-	A10N118	Okada et al., 2000
AT4G38460	*GGR*	-	-	Oh et al., 2002
AT5G47770	*FPS2*	Yes	A21N001	Delourme et al., 1994
*Core terpene synthases*				
AT1G31950	*TPS29*	Yes	-	Lange et al., 2003
AT1G33750	*TPS22*	-	-	Lange et al., 2003
AT1G48800	*TPS28*	Yes	-	Lange et al., 2003
AT1G61120	*TPS4*	Yes	-	Herde et al., 2008
AT1G61680	*TPS14*	Yes	-	Chen et al., 2003
AT1G66020	*TPS26*	Yes	-	Lange et al., 2003
AT1G70080	*TPS6*	Yes	-	Lange et al., 2003
AT1G79460	*TPS32*	Yes	-	Yamaguchi et al., 1998
AT2G23230	*TPS05*	Yes	-	Dal Bosco et al., 2003
AT2G24210	*TPS10*	-	-	Bohlmann et al., 2000
AT3G14490	*TPS17*	Yes	-	Dal Bosco et al., 2003
AT3G14520	*TPS18*	Yes	-	Lange et al., 2003
AT3G14540	*TPS19*	Yes	-	Lange et al., 2003
AT3G25810	*TPS24*	Yes	-	Chen et al., 2003
AT3G25820	*TPS27*	Yes	-	Chen et al., 2004
AT3G25830	*TPS23*	Yes	-	Chen et al., 2004
AT3G29110	*TPS16*	Yes	-	Lange et al., 2003
AT3G29190	*TPS15*	Yes	-	Lange et al., 2003
AT3G29410	*TPS25*	Yes	-	Dal Bosco et al., 2003
AT3G32030	*TPS30*	Yes	-	Lange et al., 2003
AT4G02780	*TPS31*	-	-	Mann et al., 2010
AT4G13280	*TPS12*	Yes	-	Ro et al., 2006
AT4G13300	*TPS13*	Yes	-	Ro et al., 2006
AT4G15870	*TPS1*	Yes	-	Aubourg et al., 1997
AT4G16730	*TPS2*	Yes	-	Huang et al., 2010
AT4G16740	*TPS3*	Yes	-	Fäldt et al., 2003
AT4G20200	*TPS7*	Yes	-	Lange et al., 2003
AT4G20210	*TPS8*	Yes	A21N124	Tholl and Lee, 2011
AT4G20230	*TPS9*	Yes	-	Dal Bosco et al., 2003
AT5G23960	*TPS21*	-	-	Chen et al., 2003
AT5G44630	*TPS11*	-	A21N124	Tholl et al., 2005
AT5G48110	*TPS20*	Yes	-	Dal Bosco et al., 2003

Gene abbreviations are adapted from the *Arabidopsis* Information Resource^A^.

^A^ TAIR10, www.arabidopsis.org, last accessed on December 13th, 2014.

^B^ Ohnolog pair according to Bowers et al., 2003 [8].

^C^ for a comprehensive review, see Tholl and Lee, 2011 and Phillips et al., 2008.

^D^ assosciation of *AtDXS3* to MEP pathway is subject of scientific debate (see Phillips et al., 2008).

In a next step, we therefore screened for additional members of all involved gene families within the Col-0 genome that may have been missed in previous studies in an interlaced bioinformatics approach (see Materials & Methods section). Briefly, we combined layers of information based on sequence similarity, gene synteny and Hidden Markov Modelling. First, we queried all 66 genes against the Col-0 genome in a sensitive BLAST screen (no e-value cutoff). After removing self hits, we included every target sequence for further analysis if it formed a highest-scoring sequence pair with a distant member of the initial list. This included identification of ohnologs as well as annotation and mapping of protein domains. Interestingly, this led to evidence for 19 additional genes that produce highest-scoring sequence pairs (HSPs) with genes mentioned in **Table 1**. In essence, these genes comprise tandem-, transposition- as well as ohnolog duplicate copies of genes previously known to be associated with terpenoid metabolism (**Table 2**). Note that segmental duplications are excluded from this analysis due to technical reasons (see below). Interestingly, 18 of those 19 genes are annotated as triterpene-specific synthases. Thirteen of those have been functionally characterized in previous efforts [47–49,102]. Five further ones lack functional data but have been assigned to three-letter code nomenclature in the *Arabidopsis* information resource based on computational inferences (i.e. sequence homology). Interestingly, all those loci include an encoded oxysqualene synthase domain (**S1 Table**). In contrast, one is a *DXS*-like gene with its closest homolog involved in the MEP pathway (**Table 2**). Note that the initial set of query genes applied to our first, above-mentioned BLAST analysis did not contain triterpene-specific synthases (**Table 1**). Therefore, our results indicate sequence homology of triterpene-specific synthases to genes of the core-*TPS* as well as the prenyltransferase class. Based on these findings, we hypothesize that all three groups go back to one common ancestral gene family with subsequent rounds of duplication going hand in hand with functional diversification as previously described in many cases including proline-rich proteins [103].

**Table 2:** The extended terpenoid phenotypic module in *Arabidopsis*, including triterpene- specific (C_30_) synthases.

Gene ID	Annotation	Description^A^	Tandem duplicate	Bowers pair^B^	Reference^C^
*Methylerythritol phosphate (MEP) pathway*					
AT4G15560	*DXPS2*	Desoxy-xylulosephosphatesynthase 2	Yes	A15N013	Lange et al., 2003
*Triterpene-specific synthases*					
AT1G62730	*-*	N/A; Squalene/phytoene synthase	No	-	Wang et al., 2008
AT1G66960	*LUP5*	Lupeol synthase 5	Yes	-	Herrera et al., 1998
AT1G78480	-	N/A; Prenyltransferase/squalene oxidase	Yes	-	Hanada et al., 2010
AT1G78500	*PEN6*	Pentacyclic triterpene synthase 6	Yes	-	Husselstein-Muller et al., 2001
AT1G78950	*LUP4*	Lupeol synthase 4	Yes	-	Benveniste et al., 2002
AT1G78955	*CAMS1*	Camelliol synthase 1	Yes	-	Kushiro et al., 1998
AT1G78960	*LUP2*	Lupeol synthase 2	Yes	-	Herrera et al., 1998
AT1G78970	*LUP1*	Lupeol synthase 1	Yes	-	Herrera et al., 1998
AT3G29255	-	N/A; Squalene cyclase (InterPro:IPR018333)	Yes	-	this manuscript
AT2G07050	*CAS1*	Cycloartenol synthase 1	-	-	Lange et al., 2003
AT3G45130	*LAS1*	Lanosterol synthase 1	-	-	Benveniste et al., 2002
AT4G15340	*PEN1*	Pentacyclic triterpene synthase 1	Yes	-	Husselstein-Muller et al., 2001
AT4G15370	*PEN2*	Pentacyclic triterpene synthase 2	Yes	-	Husselstein-Muller et al., 2001
AT5G36150	*PEN3*	Pentacyclic triterpene synthase 3	-	-	Husselstein-Muller et al., 2001
AT5G42600	*MRN1*	Marernal Synthase 1	-	-	Benveniste et al., 2002
AT5G48010	*THAS1*	Thalianol Synthase 1	Yes	-	Benveniste et al., 2002
*Function not clear*					
AT1G48820	*-*	N/A; tandem duplicate of *TPS28*	Yes	-	Lange et al., 2003
AT2G37140	-	N/A; best BLAST hit is *TPS1*	-	-	Lange et al., 2003

Three letter gene abbreviations are adapted from the *Arabidopsis* Information Resource^A^.

^A^ TAIR10, www.arabidopsis.org, last accessed on December 13th, 2014.

^B^ Ohnolog pair according to Bowers et al., 2003.

^C^ For a comprehensive review, see Tholl and Lee, 2011.

**Fig 2:**
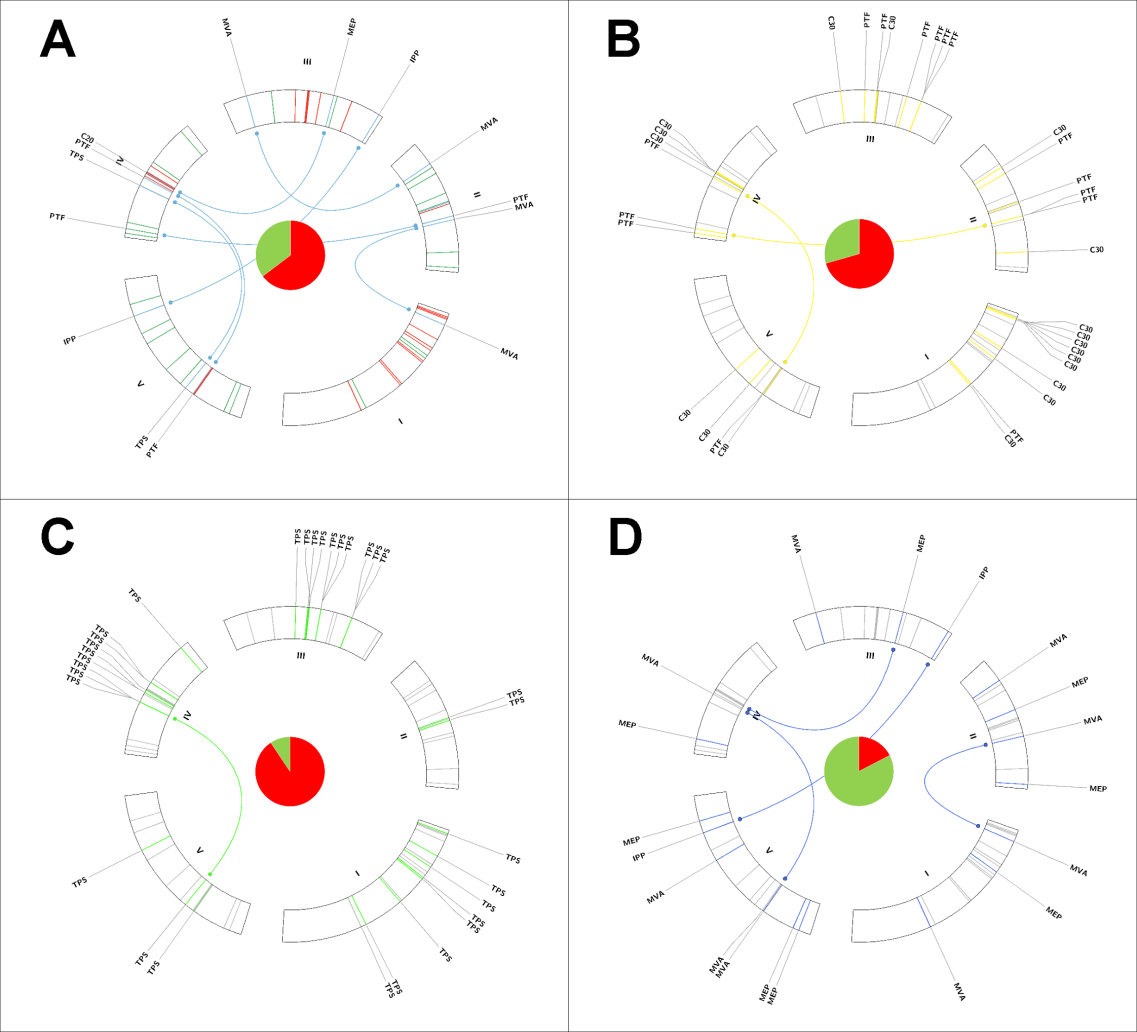
Circos ideogram showing 5 *Arabidopsis* chromosomes with the extended set of genes associated with major terpenoid biosynthetic modules. **A**. Gene inventory of the complete terpenoid biosynthetic pathway after initial expansion of published modules. Tandem duplicate supergenes are marked in red. Singletons are marked in green. Ohnolog duplicate gene pairs are marked in blue. Central pie chart shows a 68% tandem duplicate supergenes fraction. **B**. Subset of prenyltransferases and specific triterpene synthases marked in yellow. Central pie chart shows a 70% tandem duplicate supergenes fraction. **C**. Subset of core terpene synthase (*TPS*) genes marked in bright green. Central pie chart shows an 84% tandem duplicate supergenes fraction. **D**. Subset of genes associated with MEP and MVA pathways, including IPP isomerases, marked in blue. Central pie chart shows a 16% tandem duplicate supergenes fraction.

To account for the whole range of sequence diversity found among *Arabidopsis* terpenoid biosynthetic genes, we merged Table 1A and 1B and obtained pool of 85 genes putatively involved in all Col-0 terpenoid biosynthetic modules (“extended set”) (sum of all Col-0 gene entries in **S2 Table**). Initially, we dissected all members into three groups based on putative affiliation with a certain module (**Fig 1**): (a) prenyltransferases and triterpene-specific synthases, (b) core terpene synthases and (c) genes involved in MEP and MVA pathways including IPP isomerases, respectively. Visual comparison of duplicate fractions revealed striking differences between the subsets but also when comparing to the fraction of duplicates among all protein-coding genes (**Fig 2**). For the whole set of 85 genes, we found a 68%-fraction of tandem duplicate supergene clusters and a 15%-fraction of ohnolog duplicate pairs (**Fig 2A**, **Table 3 and Table 4**). For subgroup (a), we report a 70%-fraction of tandem duplicate supergenes and a 13%-fraction of ohnolog duplicate copies (**Fig 2B**). In contrast, 94% of subgroup (b) comprise members of tandem arrays, while the ohnologs fraction drops to 6% (**Fig 2C, Table 3 and Table 4**). Interestingly, subgroup (c) contains only 16% of tandem duplicate genes but a 27% fraction of genes retained after ancient polyploidy events (**Fig 2D**).

**Table 3:** Tandem Duplicates fractions among terpenoid specialized biosynthetic module in 13^A^ genomes.

Species	Genome-wide	core-TPS genes	MEP-pathway	MVA-pathway	IPP-isomerases	Prenyl-transferases	Triterpene synthases	Average^B^
*A*. *thaliana*	**15%**	94% *	-	33%	-	73% *	68% *	68% *
*B*. *rapa*	**20%**	51% *	-	7%	-	42%	40%	33% *
*T*. *hasslerania*	**17%**	62% *	18%	7%	-	53% *	20%	37% *
*C*. *papaya*	**17%**	52% *	-	-	-	-	54% *	32% *
*C*. *sinensis*	**44%**	37%	50%	56%	100%	25%	37%	**39%**
*E*. *grandis*	**33%**	73% *	18%	19%	-	60%	75% *	63% *
*G*. *max*	**73%**	42% *	12% *	-	50%	7% *	33% *	**20%**
*V*. *vinifera*	**32%**	91% *	21%	35%	-	-	96% *	78% *
*S*. *lycopersicum*	**28%**	80% *	-	13%	50%	55%	55%	56% *
*S*. *tuberosum*	**51%**	51%	-	21% *	-	-	25%	36% *
*S*. *bicolor*	**35%**	68% *	20%	-	-	14%	76% *	52% *
*Z*. *mays*	**44%**	42%	31%	40%	33%	10%	58%	**40%**
*A*. *trichopoda*	**24%**	57% *	18%	-	-	-	67%	**34%**
**Average** ^B^	**32%**	**59%**	**13%**	**17%**	**17%**	**25%**	**53%**	46% *

Minus indicates absence of tandem duplicates. Asterisks indicate significant enrichment compared to genome-wide tandem duplicate fraction based on fisher's exact test on count data (p-value threshold: 0.01). For absolute gene numbers and p-values, see S5 Table.

^A^ *C*. *Sativa*, *L*. *sativa* and *N*. *benthamiana* and *C*. *gynandraare* excluded from this analysis due to technical reasons (see Materials & Methods section).

^B^ Averages based on numbers of tandem and singleton genes, not on percentage values since gene counts in subsets are not equal.

**Table 4:** Ohnolog duplicates fractions among the terpenoid specialized biosynthetic module in 13^A^ genomes.

Species	Genome-wide	CoGe-link^C^	core-TPS genes	MEP-pathway	MVA-pathway	IPP-isomerases	Prenyl-transferases	Triterpene synthases	Average^B^
*A*. *thaliana*	22%	bit.ly/1t7DH7A	6%	13%	22%	100% *	33% *	5%	**15%**
*B*. *rapa*	53%	bit.ly/1uVTlHt	26%	54% *	73% *	100% *	74% *	33%	**49%**
*T*. *hasslerania*	48%	bit.ly/1r0khkj	26%	27%	60% *	100% *	47%	45%	**40%**
*C*. *papaya*	7%	bit.ly/1yt11Ap	-	-	-	-	17% *	-	**2%**
*C*. *sinensis*	6%	bit.ly/1xRKTJh	3%	-	22% *	-	-	-	**3%**
*E*. *grandis*	18%	bit.ly/1p2oGrm	10%	-	50% *	-	-	-	**11%**
*G*. *max*	62%	bit.ly/1yt2QNw	48%	56%	85% *	100% *	53%	27%	**57%**
*V*. *vinifera*	22%	bit.ly/1uefADr	2%	-	15%	-	40% *	-	**4%**
*S*. *lycopersicum*	19%	bit.ly/1xsTXoT	14%	-	33% *	-	-	-	**12%**
*S*. *tuberosum*	11%	bit.ly/1yWDKGZ	4%	-	17% *	-	-	-	**6%**
*S*. *bicolor*	23%	bit.ly/1xRLLxE	-	-	27% *	-	29% *	20%	**11%**
*Z*. *mays*	27%	bit.ly/11xv3rs	8%	23%	55% *	66% *	60% *	16%	28%*
*A*. *trichopoda*	7%	bit.ly/1x3SpxM	-	1%	17% *	-	33% *	-	**8%**
**Average** ^B^	**28%**		**10%**	**19%**	**42%**	**48%**	**33%**	**10%**	**18%**

Minus indicates absence of ohnolog duplicates. Asterisks indicate above-average fraction of ohnolog duplicates compared to the genome-wide background. For absolute values, see S5 Table.

^A^ *C*. *sativa* and *L*. *sativa*, *N*. *benthamiana* and *C*. *gynandra* are excluded from this analysis due to technical restrictions.

^B^ Averages based on numbers of tandem and singleton genes, not on percentage values since gene count in subsets is not equal.

^C^ Link to the CoGe platform for comparative genomics for online-regeneration of the analysis for ohnolog identification.

In summary, we found a connection to biosynthesis of mono-, di- and sesquiterpenes for 19 additional genes in *Arabidopsis* that are homologous to but absent from the published set of terpenoid biosynthetic genes. Likewise, we showed an asymmetric distribution of duplicates among genes involved in different modules of terpenoid biosynthesis in *Arabidopsis*.

### Protein domain annotation of extended genes set associated with all terpenoid biosynthetic modules in *Arabidopsis*

Increasing phylogenetic distance of plant species can lead to increased sequence diversity in homologs while the broad class of biological function remains unchanged [1,104]. For example, amino acid substitutions within the same chemical group (i.e. aliphatic, aromatic) may have little or no effects on protein function, but may result in decreased accuracy in orthologous and paralogous gene detection by sequence homology (such as BLAST) [90,105,106]. We therefore performed Hidden Markov Modelling (HMM)-driven protein motif searches and annotation among all subsets of genes involved in the extended set of Col-0 terpenoid biosynthetic genes in order to screen for additional homologs (see Materials & Methods section). Briefly, we submitted all 85 target sequences to the “Interpro5” algorithm that performs parallelized prediction of protein domains (see Materials & Methods section) [91,107–109]. This is based on machine learning for pattern recognition rather than direct sequence comparisons. As the “training” dataset for domain modelling for the submitted protein sequences, Interpro5 uses the HMM-generated profiles of all protein motif entries and associated sequences present within the pfam and various other databases [110]. Notably, benchmarking of profile HMMs and the BLAST algorithm previously revealed a higher sensitivity of HMM-based methods that is mirrored by an increased alignment quality [111].

As a result of HMM-driven protein domain annotation, we obtained a collection of all motifs encoded by all genes present in the initial set (**Table 5**). First, we pooled all terpenoid pathway-associated genes from the extended set into (a) core-TPS proteins, (b) IPP isomerases, (c) genes involved in the MEP pathway, (d) MVA pathway-associated proteins, (e) prenyltransferases as well as (f) triterpene-specific synthases and subjected all six sets to the Interpro5 algorithm [91], thereby querying a total of 14 protein motif databases. Five among those recognized motifs shared by every single member of at least 2 pools and were selected for further analysis: Interpro, Pfam, Panther, Gene3D as well as Superfamily [107,110,112–114] (**Table 5**). In a next step, we screened for protein motif entries within these 5 databases that are specific for any of the 6 aforementioned subsets of genes associated with all Col-0 terpenoid biosynthetic modules (**S1 Table**). Interestingly, our approach identified 38 domains associated with more than one subgroup due to accurate modelling of protein domain signatures (**Table 5**). Those were found either for both core-TPS proteins and prenyltransferases, or for both core-TPS genes and triterpene-specific synthases. Together with the sequence homology determined in the initial BLAST screen that formed the extended set of Col-0 target genes, this illustrates that those gene families are similar in terms of both sequence and domain structure as described above and hence might share a common evolutionary origin and function (**S1 Table**). As a result, it is not possible to affiliate one distinct homologous genes to one of these three subsets based on domain composition in every case without functional data at hand, also when utilizing specific combinations of two or more domains. In summary, we performed in-depth investigation of protein domains among all enzymes involved in every *Arabidopsis* terpenoid biosynthetic module, thereby curating a set of all detectable domains involved in the terpenoid biosynthetic pathway within the *Arabidopsis* model plant.

**Table 5:** Overview of protein domain annotation for the extended set of *Arabidopsis* terpenoid biosynthetic genes^A^.

Database	Predicted domains	Predicted domains specific for functional module	Genes with predicted domains	Genes with module-specific domains
Interpro	64	49	85	48
Panther	20	18	85	59
Pfam	25	17	85	43
Superfamily	16	10	84	11
Gene3D	16	9	83	10
**Total**	**141** (100%)	**103** of 141 (73%)	**85** of 85 (100%)	**59** of 85 (69%)

^A^ 85 target genes in the extended set of *Arabidopsis* terpenoid biosynthetic genes.

### Annotation of genes in all terpenoid biosynthetic modules across 17 target species based on both sequence homology and protein domain composition

**Fig 3:**
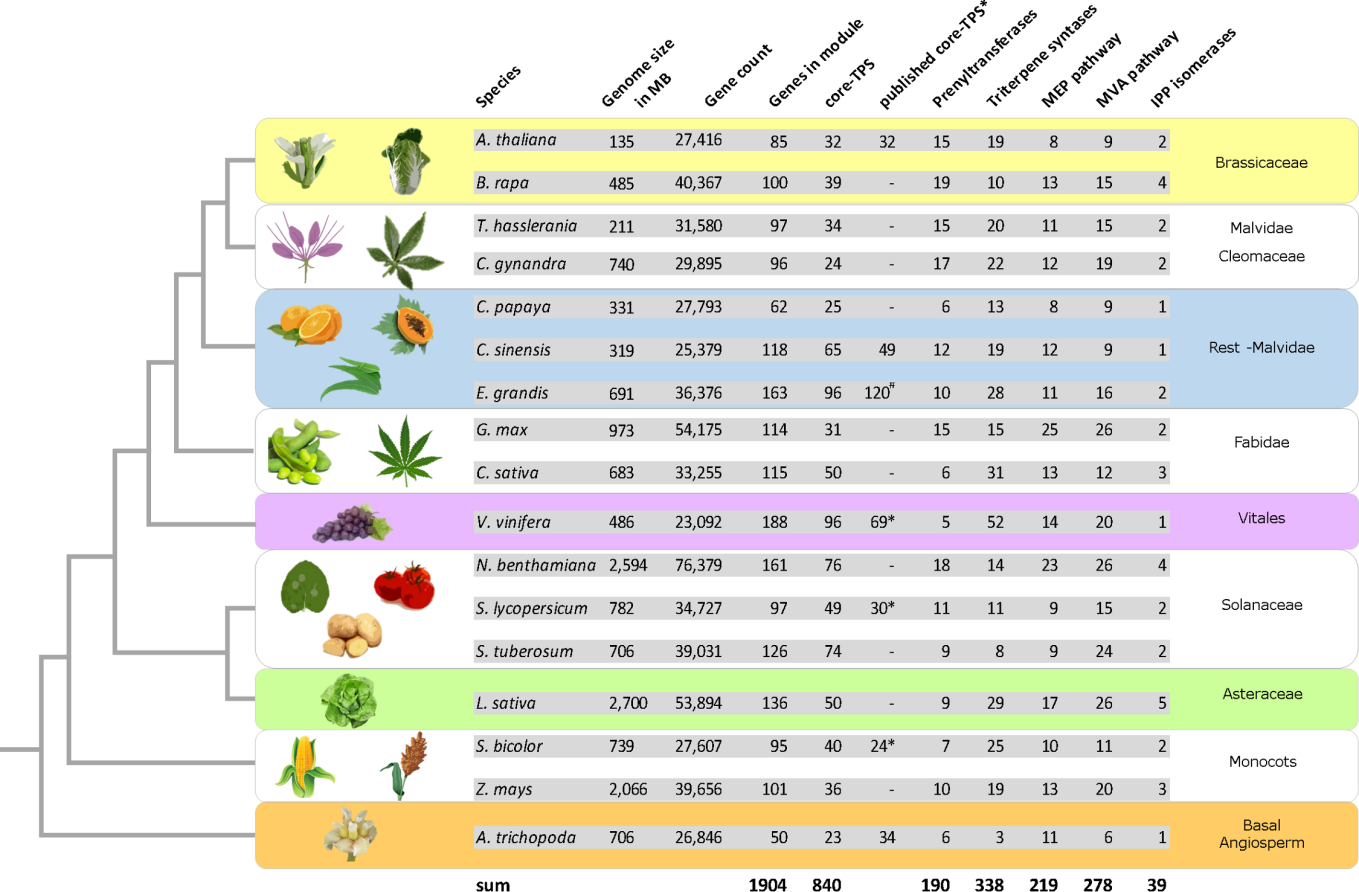
Illustration showing the complete set of genes associated with all terpenoid biosynthetic modules identified in this study across 17 genome assemblies, based on the HMM-generated profiles of Table 5. For core-*TPS* genes, numbers of previously published full-length target genes is included if available. Asterisks indicate number of previously identified full-length *TPS* open reading frames and hence putative number of functional terpene synthase enzymes. Incomplete protein fragments are not included.

We obtained a list of 1,904 protein-coding genes with putative annotation to a terpenoid biosynthetic module. To cross-reference every member to one of the six designated functional modules (**Fig 1**), we mapped all aforementioned protein motifs onto all target genes. For genes with ambiguous domain composition (i.e. presence of 38 domains without clear referencing to one functional module within the terpenoid biosynthesis, see above), we used the annotation of its highest-scoring target sequence alignment in *Arabidopsis*. Depending on sequence homology as well as on presence/absence of the aforementioned module-specific protein domains (**S1 Table**), we describe a total of 840 core terpene synthase genes (shown red in **Fig 1**), 190 prenyltransferases (shown in green in **Fig 1**), 338 triterpene-specific synthases (shown in blue in **Fig 1**) as well as 219 and 278 genes associated with the MEP (shown in purple in **Fig 1**) and MVA pathways (shown in black in **Fig 1**), respectively. Likewise, we found a total of 39 IPP isomerases (shown in turquois in **Fig 1**), summing up to 1,904 target genes in total (**Fig 3**). Please note that all sequence identifiers are appended in **S2 Table**.

Compared to the total number of protein-coding genes present in the genome, *V*. *vinifera* (grapevine) possesses the most expanded inventory of terpenoid biosynthetic genes including all modules, but also for individual modules like core terpene synthases, triterpene-specific synthases and both MEP and MVA pathways. In contrast, the highest number of prenyltransferases relative to the total number of protein-coding genes is encoded by the C4-species *C*. *gynandra*. The small gene family of IPP isomerases is most abundant (i.e. target gene count compared to number of all genes per genome) in *B*. *rapa*. Note that the *B*. *rapa* genome possesses the highest syntenic depth level among all species analyzed in this study (**S3 Table**).

In contrast, the basal Angiosperm *A*. *trichopoda* possesses the leanest inventory relative to the number of all protein-coding genes when looking at all terpenoid biosynthetic modules. Same counts for triterpene-specific synthases and for genes associated with the MVA pathway. For core-*TPS* genes, the *G*. *max* (soybean) genome encodes the smaller relative number of proteins. For prenyltransferases, we found that the *L*. *sativa* genome encodes the smallest relative number. In contrast, the MEP pathway in *S*. *tuberosum* (potato) recruits the lowest number of genes compared to all of its protein-coding genes. Finally, we found the lowest relative number of IPP isomerases within the *C*. *papaya* genome (**S3 Table**). In summary, we provide evidence for annotation of 1,904 genes to every major module of terpenoid biosynthesis within 17 target genomes, many of which have not been connected to this trait so far. Similar to functional annotation of the *Arabidopsis* genome, computational inferences of gene function comprise an important step for the future collection of functional data in wet-lab experiments [75].

### General and subset-specific cross-referencing of supergene clusters and ohnolog duplicates to terpenoid biosynthetic elements among all species

After curating a set of 1,904 target genes across 17 species, we first scored supergenes organized in tandem arrays as well as ohnolog duplicates due to polyploidy events. Second, we compared the obtained duplicate fractions between all six modules of terpenoid biosynthesis. For detection of potential enrichment or depletion of duplicate frequencies within these subsets, a species-wise comparison to the genome-wide average of tandem/ohnolog duplicates fraction was necessary. Due to technical reasons, these genome-wide fractions can’t be accurately determined for *C*. *gynandra*, *N*. *benthamiana*, *C*. *sativa and L*. *sativa* (for *Cannabis* and *Lactuca*, non-redundant RNAseq data are available only whereas the *C*. *gynandra* and *N*. *benthamiana* assemblies are highly fragmented, leading to a highly error-prone determination of genome-wide duplicates fractions, see Materials & Methods section). Therefore, our genome-wide analysis of duplicates fractions was restricted to 13 genome assemblies.

On average, 46% of all curated genes associated with terpenoid biosynthesis comprise supergenes with duplicates organized in tandem arrays. Compared to the 32% average observed for the genome-wide tandem duplicate fraction determined across the 13 genome assemblies subjected to this part of our analysis, our results highlight a significant enrichment of supergene clusters for terpenoid biosynthetic genes according to statistical analysis based on Fisher’s exact test on count data (**Table 3, S4 Table**). Next, we investigated the species-wise fractions of tandem duplicates among all identified terpenoid biosynthetic genes for comparison to the respective genome-wide background. Similar to our findings for genome-wide tandem duplicate fractions across all analyzed genomes, the significant enrichment for supergene clusters holds up for all organisms except *C*. *sinensis*, *G*. *max*, *Z*. *mays* and *A*. *trichopoda* (**Table 3**). However, comparison of duplicate frequencies within different functional modules of terpenoid biosynthesis across the 13 genomes subjected to tandem duplicate analysis did reveal certain subsets that are enriched for duplicates (five genomes were not applicable to this analysis due to technical reasons, see above). For example, triterpene-specific synthases are significantly enriched for tandem arrayed supergenes compared to the genome-wide background in *G*. *max*. Similarly, core-*TPS* genes are enriched for tandem duplicates compared to genome-wide average in the basal angiosperm *A*. *trichopoda* (**Table 3**). We have found that only *Citrus* and maize lack significant enrichment for tandem duplicates among all subsets of genes involved in terpenoid biosynthesis. Based on the enriched fraction of tandem duplicates specific for certain terpenoid biosynthetic modules, we deducted a general pattern. Both core-*TPS* genes and triterpene-specific synthases were found to be significantly more enriched for tandem duplicates across most of the analyzed species, whereas MEP and MVA pathways as well as IPP isomerase functions retained few or no supergene clusters within most analyzed species (**Table 3**, **S4 Table**).

In a next step, we determined the cumulative fraction of duplicate genes retained after ancient polyploidy events (ohnologs). Similar to the analysis of tandem duplicates, ohnolog identification relies on gene contextual information and is hence not applicable to highly fragmented gene-space assemblies or translated transcriptome datasets (see above) [4,89]. Please note that we appended URLs for online-regeneration of ohnolog identification in 13 genomes out of 17 genomes (**Table 4**). We again measured genome-wide averages wherever possible and compared them to the fractions among all subsets as described above for tandem duplicate supergenes (**Table 4**). On average, 18% of all genes associated with all modules of terpenoid biosynthesis comprise ohnolog duplicate gene copies. Compared to the 28% fraction of genome-wide ohnolog merged across all analyzed species, Fisher’s exact test on count data indicates absence of significant ohnolog enrichment for this set (**S5 Table**). In contrast, species-wise analysis revealed a significant enrichment of ohnologs among all terpenoid biosynthetic genes identified in *Z*. *mays* (**Table 4**). Moreover, analysis of species-specific ohnolog distributions among different terpenoid biosynthetic modules highlighted differential trends. In essence, we revealed patterns of above-average ohnolog retention opposite to those described for tandem duplicates. For example, dosage-independent modules like core-TPS synthases and triterpene-specific synthases contain below-average ohnolog fractions in all analyzed species (**Table 4**), while recruiting highest fractions of supergene clusters as shown above (**Table 3**).Strikingly, genes associated with dosage-dependent modules like the MVA pathway and IPP isomerases show the highest fractions of ohnolog duplicates merged across all genomes (**Table 4**). In contrast, both subsets include low fractions of tandem duplicates compared to other subsets (**Table 3**).

However, ohnolog fractions of dosage-dependent modules vary greatly between different species in many cases. The small gene family of IPP isomerases, for example, consists of 100% ohnolog duplicates within *Arabidopsis*, *Brassica*, *Tarenaya* as well as *Glycine*. In contrast, we did not detect retained ohnologs within this gene families within *Carica*, *Citrus*, *Eucalyptus*, *Vitis*, all analyzed Solanaceae as well as *Sorghum* based on the applied preferences. This is likely due to technical reasons (see Materials & Methods section). Briefly, the scoring method of SynMap depends on presence of long colinear regions and hence the N50 value indicating the “fragmentation” of the assembly. This means that false-negatives are more likely scored in genomes with many short scaffolds compared to few in the size-range of chromosome pseudo-molecules, due to the lack of information on the relative order of scaffolds.

In summary, we showed above-average fractions of ohnologs combined with below-average fractions of supergene clusters recruited by two dosage-dependent terpenoid biosynthetic modules (IPP isomerases and genes involved in the MVA pathway) (**Table 3, Table 4**). In addition, we revealed a below-average rate of ohnolog retention combined with a significantly increased rate of tandem duplicates for stoichiometrically insensitive genes (i.e. genes that are not acting in a dosage-dependent way) like core-terpene synthases as well as triterpene-specific synthases.

### Identification and phylogenetic analysis of key genes controlling isoprenoid profiles and trichome density

**Fig 4:**
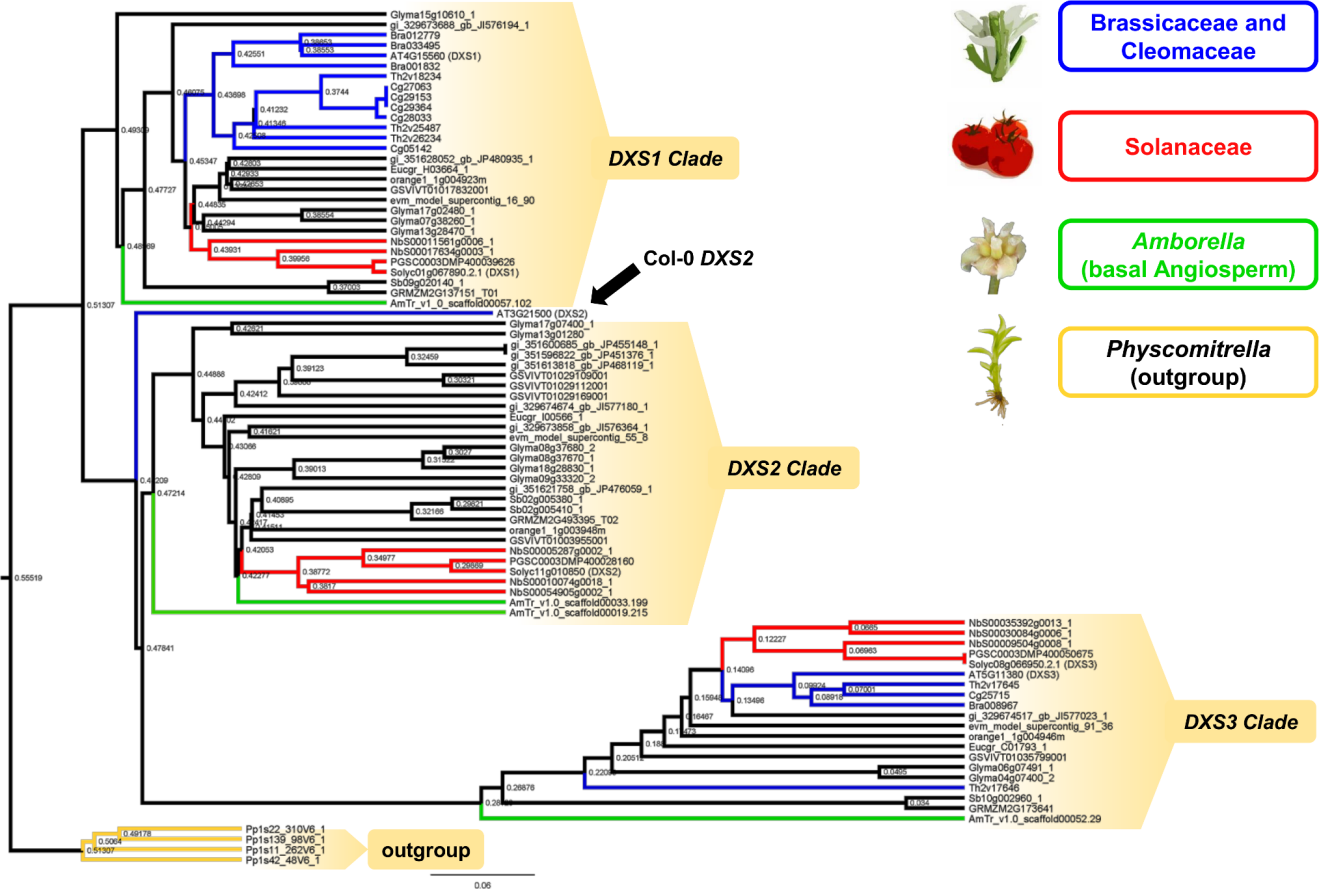
Phylogenetic relationships among 83 DXS-like proteins. Brassicaceae and Cleomaceae are marked in blue. Solanaceae are marked in red. Proteins encoded in the basal Angiosperm Amborella are marked in green. The moss *Physcomitrella* comprises the outgroup and is marked in yellow. *DXS*-like genes group in three distinct clades since the origin of Angiosperms. Notably, all analyzed Brassicaceae have lost *DXS2*-like genes. However, the model plant *Arabidopsis* contains one highly diverged member of clade two that groups closer to clade one than to any other clade two homologs (marked by black arrow).

The aforementioned biosynthetic inventory of all plant terpenoid biosynthetic modules is necessary and sufficient for production of related compounds with designated biochemical function. However, some terpenoids are autotoxic and can only be produced in high amounts in specialized hair-like aerial structures termed glandular trichomes [56,115] where they are stored or secreted to the surface in order to facilitate ecological interactions (i.e. repelling herbivores or attracting beneficial organisms). Biogenesis and distribution of trichomes is controlled by various biosynthetic and regulatory processes, often mediated by pleiotropic genes [116,117]. In this context, it has recently become evident that trichome density on the leaf surface is amongst other factors influenced by a class of pleiotropic genes that also catalyzes the entry step to the MEP pathway [35]. In tomato, two deoxy-xylulosephosphate synthase genes (*DXS*) have previously been identified. Interestingly, differential and tissue-specific expression was observed: While *DXS1* is ubiquitously expressed, *DXS2* was found to be abundant in only a few tissues including trichomes. Reduction of *DXS2* expression in cultivated tomato led to an increase in glandular trichome density [52]. To identify additional *DXS-*like homologs, we screened our curated genes set and found evidence for 79 encoded proteins within all genomes subjected to our analysis (**Fig 4, S5 Table**). In addition, we included four *DXS*-like genes that were previously identified in the moss *Physcomitrella patens* to reconstruct the evolutionary history of all 83 target genes during Angiosperm radiation [53,118].

As previously reported, the *Physcomitrella DXS*-like genes form a monophyletic clade that groups distant to all other analyzed Angiosperm target genes. Strikingly, we have identified multiple gene family members that remained un-characterized within all other analyzed annotations except maize. Within the Angiosperm clades, we found that *DXS*-like genes always group in three distinct clades that form monophyletic groups rooted by basal members present in the *Amborella* genome (**Fig 4**), which is commonly placed at or near the base of the flowering plant lineage [71,119]. Within those clades, we observed grouping of closely related species consistent with the evident phylogenetic relationships of these species as a whole (**Fig 4**). However, our analysis also revealed clade-specific differences. First, we did not detect any proteins grouping to clade three within the non-redundant *Cannabis* transcriptome (**S5 Table**). Second, whereas most organisms encode at least one member of every clade, Brassicaceae and Cleomaceae have lost *DXS*-like genes belonging to clade two (**Fig 4**, **S5 Table**). Interestingly, the model plant *Arabidopsis* forms the only exception, because it possesses one *DXS2* locus (At*DXS2*/At*DXL1* or AT3G21500) that is highly diverged from any other members present in that clade (marked by black arrow in **Fig 4**). Our analysis revealed that *AtDXS2* forms a basal sister to all other clade two members and groups closer to its clade one ortholog present in the basal Angiosperm *Amborella* compared to any other clade two members. Note that first evidence supports functional specialization at both the expression and biochemical level within the plant DXS family in *Arabidopsis (*see introduction section) [53]. In this context, the authors reveal the occurrence and putative relevance of lineage-specific gene duplications. Therefore, the plant *DXS* family emerges as an interesting model to examine the molecular evolutionary basis of plant secondary metabolism diversification, giving rise to further investigation of this gene family in a broader phylogenomics framework, as we presented in this study.

**Fig 5:**
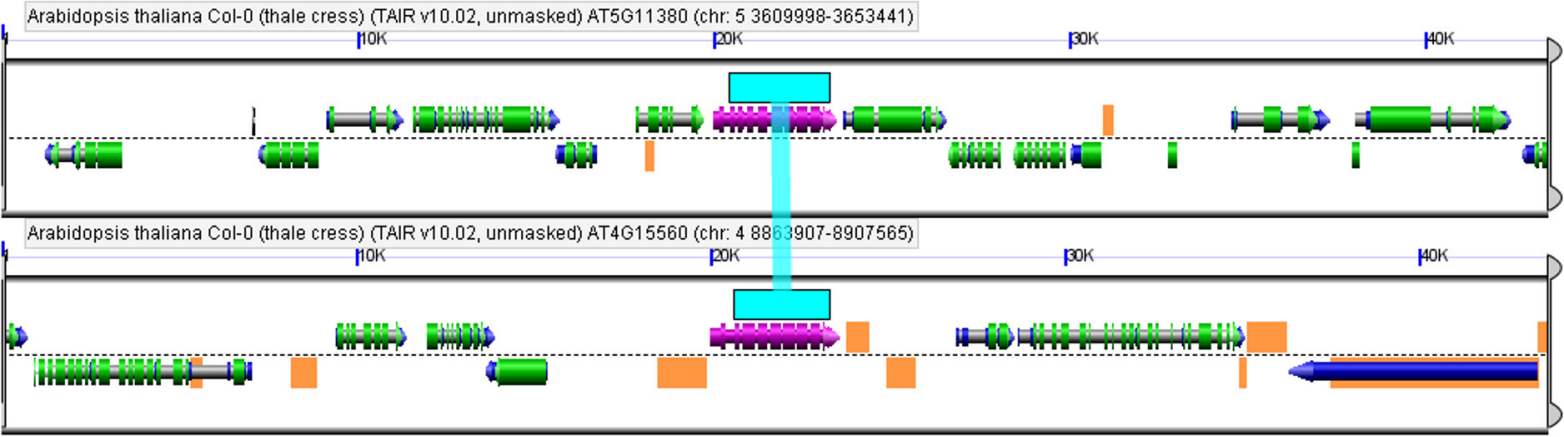
(B)LastZ two-way multiple alignment of 40kb-windows harboring the putative *Arabidopsis* gene transposition duplicate gene pair *DXS3* (AT5G11380) (upper lane, marked in purple) and *DXS1* (AT4G15560) (lower lane, marked in purple). Non-syntenic coding sequences are marked in green. Both duplicate copies form a highest-scoring sequence pair (marked in turquoise). Transposon-like sequences are marked in orange. Pseudogenes are marked in blue. Analysis can be regenerated online following the CoGe link https://genomevolution.org/r/eooq (last accessed on December 13^th^, 2014).

Next, we assessed the contribution of gene and genome duplication to *DXS*-like gene family composition among four further genome annotations with most accurate determination of ohnolog blocks (**Table 6**). To our knowledge, the contribution of genome duplication to *DXS*-like family evolution has previously not been assessed to that extend. For *A*. *thaliana*, *B*. *rapa*, *T*. *hasslerania* and *G*. max, we found 24 *DXS*-like genes in total, organized in eight duplicate groups (defined as set of genes comprising descendants from one distinct ancestral singleton due to one or more rounds of duplication) and distributed across all three *DXS*-like clades (**Fig 4**, **S5 Table**). Strikingly, 100% of those are due to ancient polyploidy events, either directly when forming pairs (WGD) or triplets (WGT) of ohnolog copies or indirectly when forming tandem- or transposition duplicates (GTD) of ohnolog group members (**Table 6**). In *A*. *thaliana*, for example, *DXS1* (AT4G15560) and *DXS2* (AT3G21500) form the ohnolog duplicate gene pair A15N013, dating back to the At-α WGD event [8,72] (**Table 1A and 1B**). The encoded proteins share 78.8% of protein sequence similarity (**Table 6**). Likewise, the corresponding genes are differentially expressed and pleiotropic (see introduction section; i.e. involved in terpenoid biosynthesis, plastid development and trichome formation [35,53,54,120,121]). Further analysis of *DXS3* (AT5G11380) indicated its putative origin due to gene transposition duplication of *DXS1*. First, both genes form a highest-scoring sequence pair based on our BLAST analysis after removal of self-hits in *Arabidopsis* (see Materials & Methods section). Second, both genes are embedded in a non-syntenic genomic regions that contain remnants of transposon-like sequences (**Fig 5**). Considering the increased phylogenetic distance between this pair of genes and its reduced degree of protein sequence similarity (**Table 6**) compared to the pair of DXS1/DXS2 (**Fig 4**), this illustrates that genetic versatility within the *Arabidopsis DXS* family was further leveraged by a gene transposition duplication (GTD). Taken together, these results give rise to the onset of functional diversification of the A15N013 ohnolog pair following the At-α WGD event in Brassicaceae (see Discussion section). Similarly, short sequence duplication may have contributed to functional diversification of *DXS*-like genes. Based on those results, we further assessed the impact of various duplication modes to all other identified *DXS*-like genes in all analyzed genome assemblies including analysis of expression and sequence diversity.

**Table 6:** Overview of gene and genome duplication responsible for *DXS*-like cluster extension; shown are all target genes for four genomes^A^.

Species	Gene Identifier	Clade	Origin of Duplication	Duplicate Group	Similarity^B^ to Duplicate copy	Identity^B^ to Duplicate copy
*A*. *thaliana*	AT3G21500	1	At-α WGD (A15N013)	1	78.8%	72.5%
*A*. *thaliana*	AT4G15560	1	At-α WGD (A15N013)	1		
*A*. *thaliana*	AT5G11380	3	GTD (AT4G15560)^C^	1	68.6%	53.3%
*B*. *rapa*	Bra001832	1	Br-α WGT^D^	2	77.9%- 81.8%	73.4%- 77.3%
*B*. *rapa*	Bra012779	1	Br-α WGT	2	92.0%	93.7%
*B*. *rapa*	Bra033495	1	Br-α WGT	2		
*B*. *rapa*	Bra008967	3	GTD (Bra033495)^C^	2	67.0%	52.6%
*T*. *hasslerania*	Th2v17645	3	Tandem (Th2v17646)^E^	3	4.3%	6.5%
*T*. *hasslerania*	Th2v17646	3	Tandem (Th2v17645)^E^	3		
*T*. *hasslerania*	Th2v18234	1	Th-α WGT^D^	4	92.4%- 93.3%	88.8%- 89.5%
*T*. *hasslerania*	Th2v26234	1	Th-α WGT	4	87.6%	83.9%
*T*. *hasslerania*	Th2v25487	1	Th-α WGT	4		
*G*. *max*	Glyma07g38260	1	*Glycine* WGD (I)	5	94.4%	91.7%
*G*. *max*	Glyma17g02480	1	*Glycine* WGD (I)	5		
*G*. *max*	Glyma15g10610	1	*Glycine* WGD (I)	5	51.7%	48.9%
*G*. *max*	Glyma13g28470	1	*Glycine* WGD (I)	5		
*G*. *max*	Glyma04g07400	3	*Glycine* WGD (II)	6	97.0%	94.3%
*G*. *max*	Glyma06g07491	3	*Glycine* WGD (II)	6		
*G*. *max*	Glyma17g07400	2	*Glycine* WGD (III)	7	45.4%	44.8%
*G*. *max*	Glyma13g01280	2	*Glycine* WGD (III)	7		
*G*. *max*	Glyma18g28830	2	*Glycine* WGD (IV)	8	96.8%	94.1%
*G*. *max*	Glyma08g37670	2	*Glycine* WGD (IV)	8		
*G*. *max*	Glyma08g37680	2	Tandem (Glyma08g37670)	8	97.6%	96.1%
*G*. *max*	Glyma09g33320	2	Segmental (Glyma08g37670)^F^	8	92.2%	86.5%
**-**	**-**	**-**	**-**	**average**	**77.6%**	**73.4%**

^**A**^ Analysis restricted to Genomes with most accurate identification of ohnologs due to technical limitation.

^**B**^ Based on encoded protein sequence.

^**C**^ Origin of GTD Duplicate based on lowest blastp e-value for alignment to other family members.

^**D**^ Embedded in most fractionated subgenome; similarity and identity scores shown relative to ohnologs in both other subgenomes.

^**E**^ Note significant length difference of both genes in this array; low similarity and identity scores indicate annotation error dividing one ORF into two neighboring genes. Both values are excluded for calculation of average.

^**F**^ Gene scored as Segmental Duplicate due to high synteny score of harbouring region while other members of duplicate group are sufficient to cover the synthenic depth of this genome (i.e. no WGT evident).

**Fig 6:**
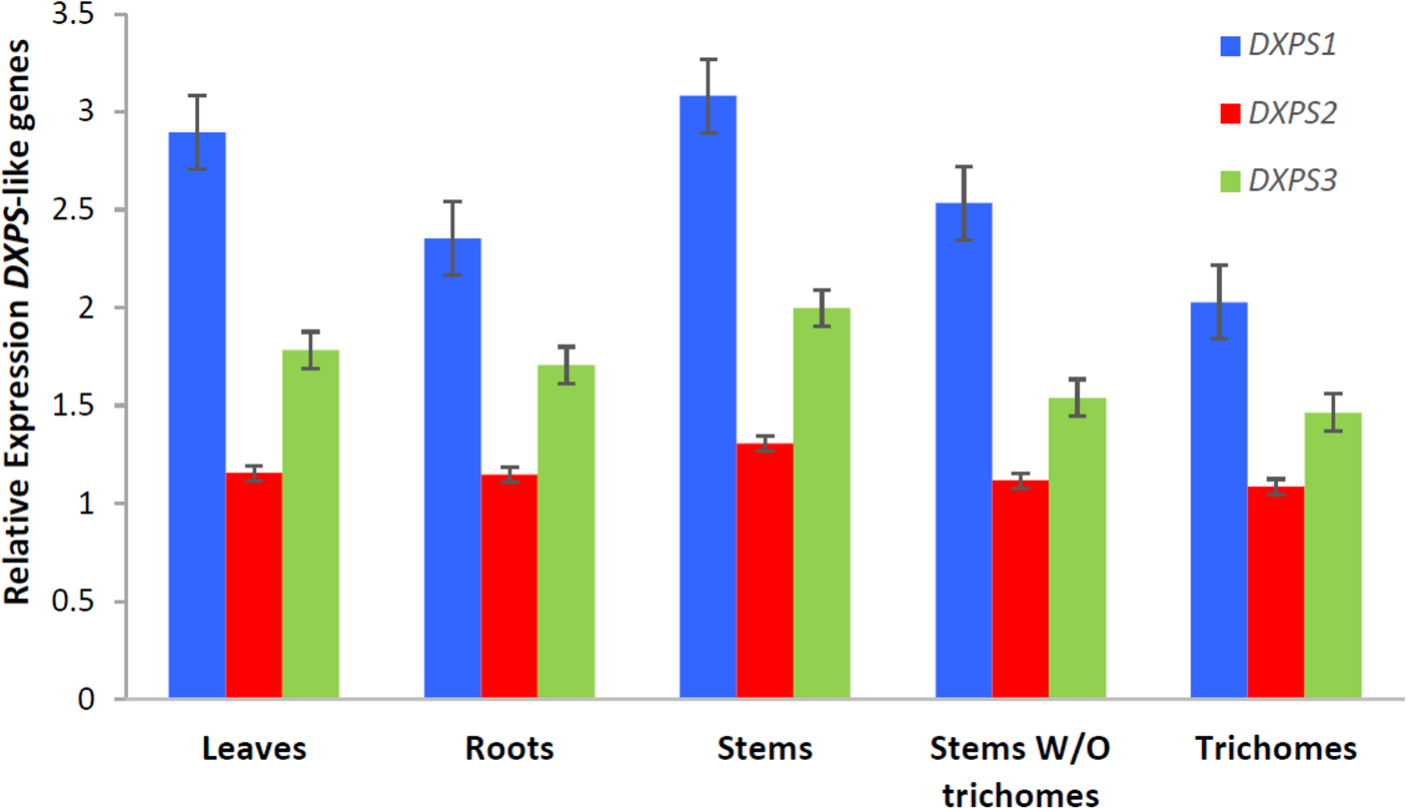
Comparative tissue-specific expression of all *Arabidopsis DXS*-like genes relative to the *bHLH* housekeeping gene. Values comprise averages of four independent ATH1 microarray experiments (Experiment ID: E-MEXP-2008, see Materials & Methods section). Notably, *DXS1* is the only member with annotation to “trichome” plant ontology (PO:0000282). The error bars represent the standard error.

Initially, we assessed divergence levels among both pairs of DXS-like protein sequences and compared those following various modes of duplication by testing for differential and tissue-specific expression of all three *DXS*-like genes in *Arabidopsis*. Please note that glandular trichomes are absent in the model plant [122]. Notably, *DXS1* is the only member of its gene family that is annotated to “trichome specific up-regulation” in the plant ontology database (PO:0000282) [123–125]. However, we confirmed expression of all three loci in *Arabidopsis* non-glandular trichomes (and various other tissue types) based on publically available microarray data [94]. Furthermore, we uncovered consistent patterns of differential expression across several tissue types. Compared to housekeeping gene expression, *DXS1* transcript are most abundant in all analyzed tissues. The ohnolog duplicate *DXS2* shows lowest expression levels, whereas the transposed duplicate *DXS3* forms an intermediate across all analyzed tissues (**Fig 6**).

To assess and compare *DXS*-like gene family divergence in further species, we have performed two separate approaches. First, we performed *DXS*-like gene expression analysis. Second, we assessed and compared the protein sequence identities of *DXS*-like duplicate groups due to different duplication events.

**Fig 7:**
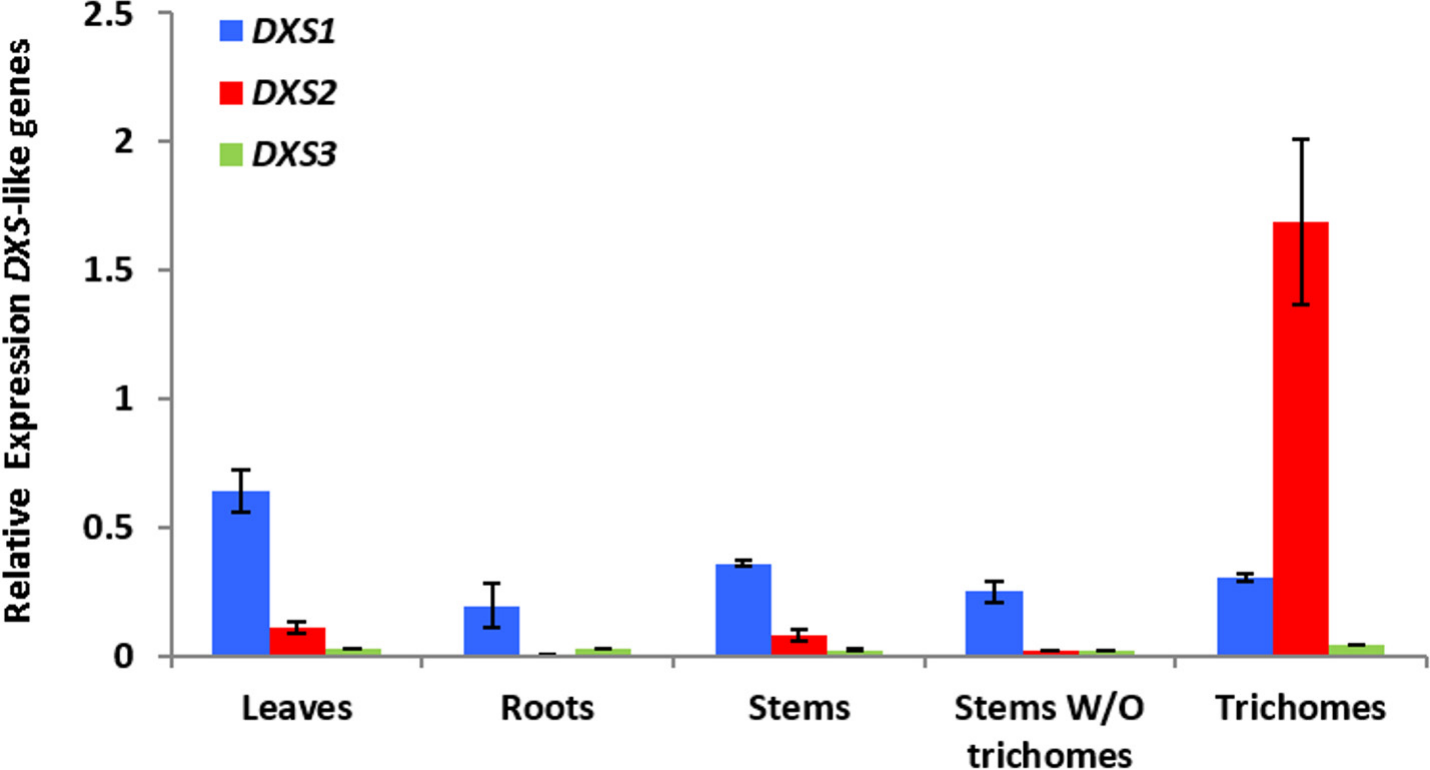
Transcript levels of *S*. *lycopersicum DXS*-like genes in different parts of the plant (leaves, roots, stems, stems without (W/O) trichomes, and isolated stem trichomes) relative to those of the reference gene *RCE1* (Solyc10g039370.1.1). Transcript levels were determined by real-time qPCR with four biological and three technical replicates for each biological sample. The error bars represent the standard error.

**Fig 8:**
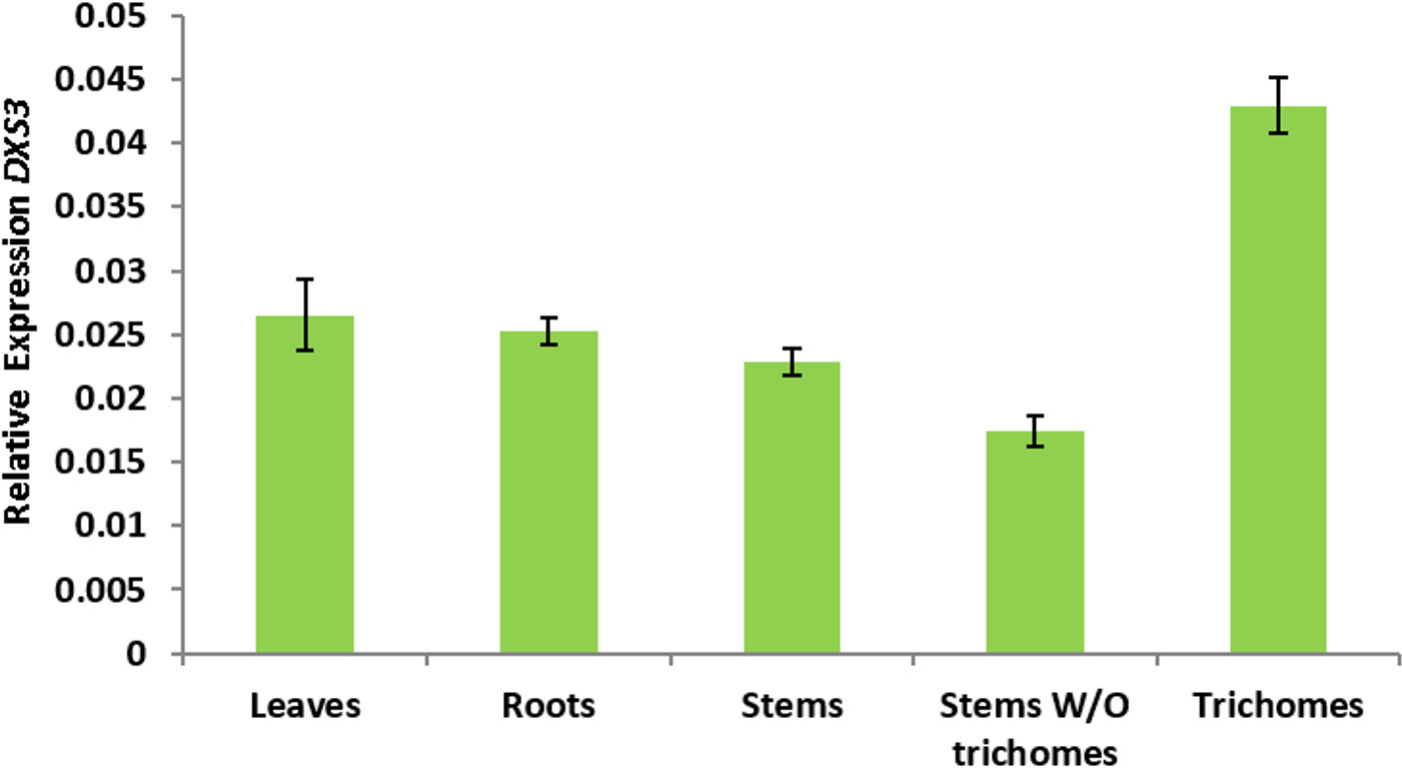
Transcript levels of the *S*. *lycopersicum DXS*3 gene in different parts of the plant (leaves, roots, stems, stems without (W/O) trichomes, and isolated stem trichomes) relative to those of the reference gene *RCE1* (Solyc10g039370.1.1). Transcript levels were determined by real-time qPCR with four biological replicates and three technical replicates for each biological sample. The error bars represent the standard error.

Gene duplication can result in transposition of the novel duplicate copy to a distant genomic location, leading to the presence of other cis-acting elements including promotors or enhancers that influence gene expression [126,127]. This results in sub-functionalization of segregants on the expression level. To extend the aforementioned findings concerning sub-functionalization of *DXS* genes in *A*. *thaliana*, we have tested expression of *S*. *lycopersicum* target genes in every clade. In addition to increased expression of *DXS2* in trichomes and global expression of *DXS1* that was previously made evident [52] (**Fig 7**), we have uncovered that transcript levels of *DXS3* are almost 2-fold higher in trichomes compared to any other analyzed tissue type (**Fig 8**).

In addition to frequent changes in gene expression, recent analysis revealed an accelerated rate of amino acid changes when comparing ohnolog duplicates to their paralogs [128]. High rates of amino acid substitutions lead to decreased levels of protein sequence identities when comparing gene copies due to different duplication modes. For example, polyploidy facilitated rapid diversification of protein sequences and sub-functionalization on a biochemical level in several cases, including glucosinolate biosynthesis, resistance proteins of the NB-LRR type as well as L-type lectin receptor-like kinases [25,26,129]. In all three cases, functional diversification among certain duplicate pairs correlates with differentially decreased protein sequence identities when comparing “novel” gene copies due to certain duplication events. Therefore, we assessed protein sequence similarity/identity among all other seven *DXS*-like duplicate groups (i.e. sets of genes due to duplication of one distinct ancestral singleton), thereby screening for indications of putative sub- or neo-functionalization (**Table 6**). Values for protein sequence similarity (identity) range from 45.4% (44.8%) (*G*. *max*, duplicate group 7) to 96.8% (94.1%) (*G*. *max*, duplicate group 8). In summary, DXS-like proteins share an average of 77.6% (73.4%) for sequence similarity (identity) among all groups, thereby reaching a cumulative divergence level similar to that observed in *A*. *thaliana*, for which data on differential target gene expression following gene and genome duplication are available (see above).

In summary, we have analyzed three clades of *DXS*-like genes present in every analyzed genome annotation. We have assessed differential and tissue-specific expression for two distant lineages, thereby collecting indications for putative sub-functionalization following gene and genome duplication within this group of target genes. To further support this hypothesis, more sequence and expression data are necessary from basal angiosperms in order to facilitate comparison of the observed profiles in a more ancestral state.

## Discussion

### A combination of synteny, sequence similarity and protein domain modelling facilitates large-scale gene identification and novel annotations in all modules of terpenoid biosynthesis

In a genome informatics approach, we combined a novel and easy-to-follow meta-method for gene and supergene cluster identification with a custom pipeline for *de novo* protein annotation for large-scale identification of biosynthetic elements associated with plant secondary metabolism. The method provided in this article is novel because it integrates information provided by the genomic location of target genes to information on sequence homology and to the information on encoded protein domain composition. Our method can be applied to a collection of final stage genome annotations, early-stage gene-space assemblies as well as non-redundant transcriptomes, thereby facilitating uniform standards for gene identification. In this context, we analyzed various datasets of different quality for a flowering-plant wide comparative survey of genes building up a major pathway of plant specialized metabolism. In summary, we curated a set of genes associated with all modules of terpenoid biosynthesis and determined key factors shaping metabolic diversification in an Angiosperm-wide scale.

First, we investigated 17 species including twelve major crops. During this initial part of our analysis, we discovered previously uncharacterized genes of the (a) *TERPENE SYNTHASE*- as well as the (b) *DXS*-like types in all species except *Arabidopsis* and *Eucalyptus*. These gene families have often been subjected to species-specific analysis in the past because they are involved in (a) generating a diverse set of terpenoid compounds and (b) in control of trichome density on the leaf surface, thereby providing significant economic and ecologic potential. The provided data on novel annotations of target genes in most species elucidated the power of our approach in a proof-of-concept and may act as a blueprint for future efforts to more rapidly find and clone functional core-*TPS* and *DXS*-like genes from any flowering plant in context of plant breeding and biotechnology.

Second, we identified various genes that have previously not been associated with a distinct function and established computational inferences to encoded prenyltransferases and triterpene-specific synthases across all lineages. These enzymes are commonly associated with the biosynthesis of di-, sesqui-, tri-, tetra- and polyterpenes. Assessing similarities to core-*TPS* genes in both coding sequence on the DNA level and protein domain composition, we provided indications for the common evolutionary origin shared among all three gene families. Furthermore, we monitored the underlying variation of gene copy number in a phylogenomics framework and thereby described a framework that increased genetic versatility to create the necessary basis for metabolic diversification within a timeframe of 250 MA corresponding to flowering plant radiation.

Third, our approach identified homologs of all genes currently annotated to MVA and MEP pathways including *DXS*-like genes in *Arabidopsis* across all analyzed genomes. Large-scale annotation of genes employed by those pathways has to date not yet been made available for every analyzed species except *Arabidopsis*, tomato and potato. In this context, our study provides and important prerequisite for future efforts aiming at metabolic engineering within any of the analyzed crop lineages.

### Both gene- and genome duplication mediated a dramatic increase of genetic versatility underlying modular terpenoid biosynthesis in all species

In the next part of our analysis, we screened for gene and genome duplication events that affected copy number of all loci involved in distant modules of terpenoid biosynthesis across all investigated species. In this context, genetic versatility is defined as the number of homologs within one gene family. Including novel annotations of previously un-identified genes to all six modules (see above), we described a 376%-increase of terpenoid biosynthetic gene copy number (“genetic versatility”) ranging from the leanest state found in the basal Angiosperm *Amborella* (50 genes) up to most versatile genotypes found for *Vitis* that has been subjected to extensive human domestication (188 genes). Merging the genetic inventory associated with all six modules, we revealed that this increase is driven by a combination of gene and genome duplication across 250 MA corresponding with the radiation time of flowering plants. However, individual differences apply when considering single terpenoid biosynthetic modules separately. To our knowledge, this is the to-date most intensive and systematic study of plant gene family expansion that influenced metabolic diversification in a phylogenomics framework.

Please note that segmental duplications are excluded from our analysis. In this context, we acknowledged an error rate due to false-positive scoring as ohnolog duplicates affecting ancient segmental duplications of large genomic regions. Briefly: It is currently not possible to accurately distinguish large segmental duplications from fractionated blocks due to genome multiplications in all cases. Likewise, very short segmental duplications with high degree of fractionation may be accidentally scored as a series of distant gene transposition duplication. This is mainly due to technical reasons, because the SynMap algorithm controls scoring of synteny merely based on a function of collinear genes in a certain density as previously described [4,87,88]. However, most segmental duplications that did not emerge roughly at the same time than any of the investigated genome duplications will display significantly different averages for ka/ks values, and are therefore excluded from synteny analysis due to the cut-offs thresholds applied in the SynMap preferences (see Materials & Methods section).

### Enzymes catalyzing the committed step of end product biosynthesis are more often encoded by supergene clusters due to tandem duplication

We highlighted a consistently asymmetric distribution of supergene clusters across all terpenoid biosynthetic modules. Generally, core terpene synthases as well as triterpene-specific synthases comprise enzymes catalyzing the committed step for biosynthesis of designated end products (mono-, di-, sesqui-, and triterpenes). We revealed that those are most enriched for tandem duplicate copies across all analyzed genomes. Please note that in alignment with this these findings, the role of syntenic core-*TPS* supergene clusters that include adjacent loci involved in different modules was recently made evident for diversification of terpenoid pathway assembly during radiation of various Angiosperm clades (see below) [33]. Moreover, it has become evident that single-featured polymorphisms affecting those genes are sufficient to alter, amongst others, herbivore behavior in otherwise isogenic lines [130–132]. In the opinion of the authors, such processes may have correlated with human efforts of plant domestication and crop breeding in multiple cases. It seems possible that sub-functionalization following tandem duplication of target genes influenced key traits (i.e. scent, taste), making the plant more suitable for further selection. This hypothesis is supported by the high target gene count for highly domesticated species with high content of terpenoids (like *Vitis*, *Cannabis* and *Lactuca*). Although *Eucalyptus* possesses the highest terpenoid biosynthetic gene count among all species analyzed in this study, it did not undergo major processes of human domestication [78]. However, several herbivores are known to respond differently to *Eucalyptus* inter- and intraspecific variation of secondary metabolite profiles with potential effects on target gene evolution [133]. Please also note that intensive domestication may also lead to a low *TPS* gene count in some cases, for example as a result of selection towards different key traits negatively influenced by genes in linkage disequilibrium to *TPS* genes [134].

### Dosage-dependent enzymes in modules mediating intermediate reaction steps are more often encoded by ohnolog duplicates–Introducing a two-step model for rapid plant pathway diversification

Compared to the above-mentioned asymmetric distribution of tandem duplicate copies across all subsets of genes involved in terpenoid biosynthesis, we reported opposite tendencies for retained ohnologs. We made evident that multi-gene family members involved in the MVA pathway as well as IPP isomerases more often tend to originate from whole genome multiplication events. For the MVA pathway, ohnolog fractions greatly outreach genome-wide averages for all genome annotations except papaya. IPP isomerases comprise 100% of retained ohnologs in Brassicaceae, Cleomaceae as well as *Glycine*. These groups of gene copies are due to duplication of a distinct ancestral singleton (“duplicate groups”) but encode enzymes involved in different terpenoid functional modules, working together by catalyzing neighboring reactions and isomerization of intermediate products (IPP or MVA/MEP modules). According to the gene balance hypothesis, duplicate loci are preferentially retained when functioning together in a dosage-dependent way [6,135]. In this context, we showed an asymmetric ohnologs distribution among the modules acting up- and downstream of core terpene scaffold synthesis.

Based on those findings, we hypothesize a two-step mechanism for the rapid plant pathway- and trait diversification observed in nature. This proposed mechanism depends on both gene- and genome duplication and affects different groups of genes at different times. In a first step, ancient polyploidy plays a paramount role by mediating the described expansion of certain genetic networks involved in plant primary metabolism (like MEP/MVA and *IDI* loci, see **Fig 1**), thereby creating a certain degree of “pathway redundancy”. Due to stoichiometric effects, the following post-polyploidy rate of plant survival depends on parallel retention of most (if not all) duplicated genes present in affected metabolic modules. Both functional diversification of ohnolog duplicates and/or incomplete module retention may lead to detrimental effects due to altered fractions of primary metabolite concentrations, as previously hypothesized and backed up by gene network analysis in context of mustard family evolution [135,136]. In a second step, more recent, short sequence duplications (including tandem and gene transposition duplication) creates an extended pool of trans-acting elements (like, for example, additional core-*TPS* or *DXS* genes). Since increased copy number of those genes does not lead to detrimental effects due to stoichiometry as described above, functional diversification may create extended capabilities to catalyze biosynthesis of extended product ranges (novel functions). The aforementioned polyploidy-induced primary module duplication created a superabundance of primary metabolites, thereby providing a “playground” for the evolution of novel functions catalyzed by novel gene copies due to short sequence duplicates.

In a nutshell, our results provided evidence for a partial polyploidy-driven expansion of plant secondary metabolism and strongly supported the gene-balance hypothesis for the dosage-dependent subset of involved key genes. Such trends have often been suggested for plants [14,23,137], but solid evidence on a genetic level was to-date only available for glucosinolates and plant resistance proteins of the NB-LRR type [25,26].

### Duplicate gene copies of ancestral singletons diversified in metabolic function following gene and genome duplication: the case of *DXS*-like genes

Recent analysis strongly support the concept of functional specialization following gene duplication as the evolutionary fate explaining retention of the duplicated gene pair DXS1/DXS2 in *Arabidopsis* [53]. Based on this approach, we performed follow-up analysis of *DXS-*like gene family evolution on a broader phylogenomics scale. In summary, we showed that certain sets of duplicate gene copies that descend from duplication of one ancestral singleton (i.e. duplicate groups) contain genes encoding different enzymes for the same pathway in *Arabidopsis* and tomato. Some of those convey pleiotropic effects due to published annotation to different traits (i.e. control of trichome density and terpenoid biosynthesis). Additionally, we identified common protein motifs present (a) within and (b) across different modules of terpenoid biosynthesis. We conclude an expansion of isoprenoid pathways by gene family diversification following gene and genome duplication, thereby resulting in the complex, modular architecture of terpenoid biosynthesis and the plethora of produced compounds observed across the Angiosperm clade. Because supergene clusters tend to be younger than genes preferentially retained after ancient polyploidy events [11,138], ohnologs are likely prone to acquire additional roles over time as previously described (sub- and neo-functionalization) [135,139,140].

Moreover, we have found evidence for the preferential (i.e. above-average) retention of *IPP* genes following various independent, successive polyploidy events for the Brassicaceae-Cleomaceae sister group system [83]. Similar to DXS-like proteins, IPP isomerases convey pleiotropic functions because they are relevant for the biosynthesis of other isoprenoid compounds beyond plant terpenoid biosynthesis. They also have been brought in connection with plant development in *Arabidopsis*, thereby mediating a check-point for primary metabolism (e.g. hormones) and different branches of specialized metabolism [141–143]. The observed trend of *IDI* over-retention is consistent for species-specific WGT events (Th-α for *Tarenaya* and Br-α for *Brassica*) as well as for the more ancient At-α WGD event shared by all Brassicaceae [8,17,72,76,144]. Similarly, we observed a rising *IDI* gene counts following soybean polyploidy. We concluded a preferential retention of this gene family following polyploidy that might be due to reported dosage-sensitivity (see Introduction section) and is likely visible especially in the aforementioned genomes due to their high levels of syntenic depth (i.e. high levels of genome multiplicity due to more successive WGDs/WGTs compared to other genomes). However, the case of *Arabidopsis* provides an exception which might be due to its reductive genome state that has been previously reported for the genus of the model plant [145].

Furthermore, our results further support the concept of sub-functionalization among *DXS*-like genes on a broader phylogenomics scale than previously reported [53]. In addition, we assessed and compared the differential impact of various duplication modes (i.e. WGD and short sequence duplication) to functional diversification of *DXS*-like genes, thereby uncovering novel aspects shaping target gene family evolution. Similar to *IDI* loci, *DXS*-like genes have been associated with more than one trait. Two among three *DXS*-like genes in *Arabidopsis* comprise the retained ohnolog pair A15N013, dating back to the At-α that is shared by all Brassicaceae. While both *DXS1* (AT4G15560) and *DXS2* (AT3G21500) are annotated to the MEP pathway, *DXS1* is also involved in plastid development [8,52,72,120,121]. In addition to the reported control of isoprenoid profiles, functional evidence for control of trichome density on the leaf surface has been made evident [52]. Initially, we discovered a whole new clade of *DXS*-like genes with members in Solanaceae and Brassicaceae including *Arabidopsis*. Next, we scored the contribution of ohnolog retention to the set of target loci identified the Brassicaceae-Cleomaceae sister group system as well as the legume *G*. *max*. We showed that all target genes within the aforementioned four genome annotations date back to ancient polyploidy events, either directly by comprising ohnolog duplicate groups or indirectly by comprising tandem- or transposition copies of ohnologs. Furthermore, we unraveled phylogenetic relationships within the target gene family that groups to three clades of encoded DXS-like proteins. We brought those clades in connection with a expression polymorphisms following gene- and genome duplication in tomato and the model plant *Arabidopsis*, thereby elucidating another case of putative sub-functionalization following duplication.

### Modified terpenes: Future work or going beyond the plant terpenoid biosynthetic module

Recently, Boutanaev et al. published a very conclusive investigation of core-*TPS* gene diversification across an evolutionary timeframe similar to the scope of our study (see introduction) [33]. The authors defined an (incomplete) “terpenome” that merely consists of (some, but not all present) core-*TPS* genes and supergene clusters that consist of both core-*TPS* and *CYP*-like genes. *CYP*-like genes encode cytochrome P450 enzymes that catalyze downstream modifications of various secondary metabolite core structures including alkaloids, glucosinolates and terpene post-modification reactions [146–148]. The authors infer an important role of (micro)synteny and *TPS*/*CYP-*locus linkage disequilibrium for terpenoid pathway assembly in plants, and suggest a differential mechanism of trait diversification in monocots and dicots [33]. However, terpenoid biosynthesis in plants is modular because it consists of more than just the core-*TPS* gene family (**Fig 1**). Likewise, *CYP*-like genes are not the only family mediating terpene post-modification reactions [35]. Due to our large-scale annotation of terpenoid biosynthetic genes across all pathway modules within 17 representative genomes, our results provide a valuable basis for future efforts to further investigate the role of synteny and genetic linkage disequilibrium in context of a more complete “terpenome”. This includes the possibility to better elucidate the effects of genetic co-segregation with many other gene families that convey terpene downstream modifications, similar to the aforementioned case study published by Boutanaev et al. [33]. Such gene families may include, for example, UDP glucuronosyltransferases and many other pleiotropic genes involved in biosynthesis of terpenoids and, beyond that, various other plant secondary metabolites [149]. Ultimately, the data provided in our study will facilitate a better understanding of plant secondary metabolite pathway assembly in Angiosperms with various implications for plant breeding and metabolic engineering in context of medicine, flavor, fragrance and pigment production.

## Supporting Information

S1 Table HMM-driven protein domain prediction among the extended set of *Arabidopsis* terpenoid biosynthetic genes. (XLS)

S2 Table Cross-referencing of 1, 904 target genes among 17 genomes to a specific subset of genes acting in the terpenoid biosynthetic module. (XLS)

S3 Table Species-specific relative size of terpenoid biosynthetic modules. Numbers are quotients of the module-wise gene count of terpenoid biosynthetic pathways and the number of all protein-coding genes within the whole genome. Species with highest and lowest relative pathway size among all analyzed species are color-coded as indicated in the legend.
(XLS)

S4 Table Species-wise distribution of *DXS*-like genes among three subgroups. (XLS)

S5 Table Comparison of genome-wide numbers of tandem/ohnolog duplicates to numbers among subsets of the terpenoid biosynthetic module, including p-values from Fisher's exact test on count data. Red indicates absence of tandem/ohnolog duplicates. Green indicates significant enrichment among terpenoid biosynthetic genes compared to background with threshold of 0.01.
(XLS)
